# Synthesis, Kinetics, Binding Conformations and Structure-activity Relationship of Potent Tyrosinase Inhibitors: Aralkylated 2-aminothiazole-ethyltriazole Hybrids

**DOI:** 10.22037/ijpr.2020.15521.13145

**Published:** 2021

**Authors:** Abdul Rehman Sadiq Butt, Muhammad Athar Abbasi, Aziz-ur- Rehman, Sabahat Zahra Siddiqui, Hussain Raza, Mubashir Hassan, Syed Adnan Ali Shah, Sung-Yum Seo

**Affiliations:** a *Department of Chemistry, Government College University, Lahore-54000, Pakistan. *; b *College of Natural Sciences, Department of Biological Sciences, Kongju National University, Gongju, 32588, South Korea. *; c *Institute of Molecular Biology and Biotechnology, The University of Lahore, Lahore, Pakistan. *; d *Faculty of Pharmacy and Atta-ur-Rahman Institute for Natural Products Discovery (AuRIns), Level 9, FF3, Universiti Teknologi MARA, Puncak Alam Campus, 42300 Bandar Puncak Alam, Selangor Darul Ehsan, Malaysia.*

**Keywords:** Thiazole, Triazole, Aralkyl halides, Tyrosinase, Kinetics, Molecular docking

## Abstract

Considering the diversified pharmacological importance of thiazole and triazole heterocyclic moieties, a unique series of *S*-aralkylated bi-heterocyclic hybrids, **7a-l**, was synthesized in a convergent manner. The structures of newly synthesized compounds were characterized by ^1^H-NMR, ^13^C-NMR, IR, and EI-MS spectral studies. The structure-activity relationship of these compounds was envisaged by analyzing their inhibitory effects against tyrosinase, whereby all these molecules exhibited potent inhibitory potentials relative to the standard used. The Kinetics mechanism was ascertained by Lineweaver-Burk plots, which revealed that **7g** inhibited tyrosinase non-competitively by forming an enzyme-inhibitor complex. The inhibition constants K_i_ calculated from Dixon plots for this compound was 0.0057µM. These bi-heterocyclic molecules also disclosed good binding energy values (kca**l/**mol) when assessed computationally. So, these molecules can be considered promising medicinal scaffolds for the treatment of skin disorders.

## Introduction

Heterocyclic compounds are the main class of organic chemistry. They are enormously used in the biological and industrial fields. Heterocycles are a vital part of all well-known organic compounds which are used in material sciences, optics, and pharmacology. Heterocyclic nucleus plays a significant role in medicinal chemistry and functions as a key template for the development of various therapeutic agents (1). The heterocyclic compounds such as *mono*-azo compounds composed of sulfur and/or nitrogen atoms are widely used as building blocks in chemistry and are biologically active, containing a broad range of activities (2, 3).

Thiazole is a five-membered heterocyclic compound having both sulfur and nitrogen atom in the ring. This ring also exists in different significant natural products like penicillin and vitamin B1 (thiamine) (4). 2-Aminothiazole is a typical heterocyclic amine used to synthesizevarious compounds, including dyes, fungicides, sulfur drugs, reaction accelerators, and as an intermediate for the synthesis of antibiotics. Furthermore, due to provision of substitution with different groups, a large number of 2-aminothiazoles are being used for pharmaceutical purposes (5-7). Such derivatives continue to attract the attention of biologists by virtue of their use in the treatment of biological systems. These thiazole, containing heterocycles have been reported to act as anti-leukemic (8), antimicrobial (9-11), antiproliferative (11), anti-inflammatory, antifungal (12), antiviral (7), anesthetic (13), and enzyme inhibitor (14). The compounds containing triazolyl clubbed with thiazole have been reported as potential agents for the treatment of Alzheimer’s disease (15).

In recent years, for drug discovery, compounds containing triazole rings have become potential targets. The significance of triazole derivatives lies in their varied biological activities and they have attained a unique position in heterocyclic chemistry (16, 17). Among various triazoles, 1,2,4-triazole core has acquired a superior position owing to its wide-ranging variety of bioactivity (18). 1,2,4-triazole nucleus is the essential part of many therapeutically important agents. Vorozole, Letrozole, and Anastrozole (antitumor) (19), Ribavirin (antiviral) and Rizatriptan (antimigraine) (20) are few examples of drugs having a 1,2 ,4-triazole moiety in their structures. Itraconazole, Posaconazole, and Fluconazole are effective antifungal drugs used in current treatment (21, 22). *N*-substituted triazole attached with several heterocyclic nuclei have been associated with various biological activities such as antioxidant (23), antibacterial, antifungal (24, 25), antiplatelet (26), analgesic, anti-inflammatory (27), potassium channel activators (28), anticonvulsant (29), anticancer (30), antiviral (31) and antitumor (32, 33).

Tyrosinase is found in many mammalian, plants, and fungi cells. It is extracted from the *Agaricus bisporus,* a champignon mushroom and is homologous with that of mammalian. As mushroom tyrosinase is commercially available, so almost all the studies of tyrosinase inhibition have used mushroom tyrosinase. The name of the tyrosinase was given due to the activity of tyrosine amino acid, which is found in almost all animal cells and plays a very important role in synthesis of melanin (34). Human tyrosinase is a single membrane-spanning transmembrane protein, and it catalyzes the first two reactions of melanin synthesis, the hydroxylation of L-tyrosine to L-3,4- dihydroxyphenylalanine, L-DOPA, and the oxidation of L-DOPA to dopaquinone. Dopaquinone spontaneously forms an orange-red pigment called dopachrome, which undergoes a final reaction to form the blackish- brown pigment melanin. In Stage 1 of skin cancer (melanoma), the enzyme is hardly noticeable, but is widespread and evenly distributed in Stage 2, and then unevenly distributed in Stage 3 (metastases) (35).

Melanin is present in plants, bacteria, fungi, and keratinocytes of hair and skin of animals. It is catalyzed by tyrosinase which plays a critical role in determining skin, eye, and hair color. It also played a very important role in preventing overheat of internal organization and protecting the eye and skin from ultraviolet radiation (36, 37). Melanin content is also responsible for the browning of food. Inborn absence or defect of tyrosinase causes a disorder of melanin production in the body called albinism. Overactive tyrosinase produces excess melanin, which is also linked to skin disorders. Abnormalities in tyrosinase activity may also cause Parkinson’s disease (38, 39).

In current years, tyrosinase studies are mainly on albino, melanoma, pigment obstructive disease, and early-onset Alzheimer’s disease (40). Therefore, it is necessary to obtain new tyrosinase inhibitors from altered sources. Arbutin, azelaic acid, kojic acid and hydroquinone are used as tyrosinase inhibitors in pharmaceuticals, and cosmetics (41-43). But due to cytotoxicity, mutagenesis and irritation effects, the use of hydroquinone is prohibited (44). On the other hand, due to poor skin penetration, very low *in-vivo* efficacy, and unsatisfactory formulation stability of arbutin and kojic acid, their use is also limited (45). So, there is a need to search new, safer, and proficient tyrosinase inhibitors to trat pigment disorders (46, 47).

**Figure 1:**
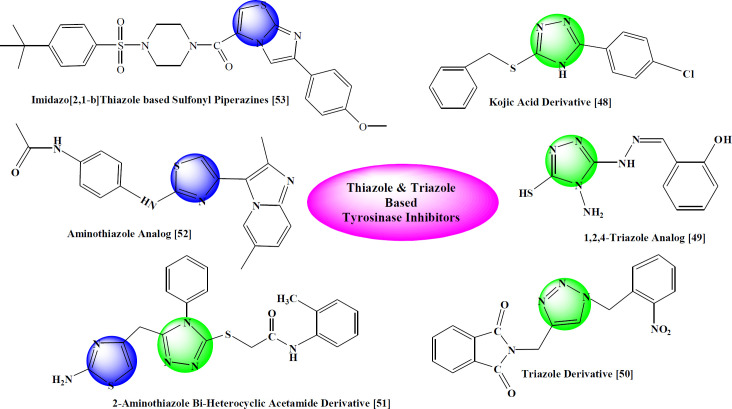
Rationale of the current study

Previously some thaizole and triazole derivatives shown in Figure 1 have been reported as tyrosinase inhibitors (48-53), so the present investigation was rationalized to explore the therapeutic potential of new thiazole-triazole hybrid molecules as tyrosinase inhibitors to overwhelm the problems of skin disorders.

## Experimental

*Chemistry*

Chemicals were purchased from Sigma Aldrich and Alfa Aesar (Germany), and solvents of analytical grades were supplied by local suppliers. By using the open capillary tube method, melting points were taken on Griffin and George apparatus and were uncorrected. Using thin layer chromatography (with ethyl acetate and *n*-hexane (30:70) as mobile phase) the initial purity of compounds was detected at 254 nm. Elemental analyses were performed on a Foss Heraeus CHN-O-Rapid instrument and were within ± 0.4% of the theoretical values. IR peaks were recorded on a Jasco-320-A spectrometer by using the KBr pellet method. EI-MS spectra were measured on a JEOL JMS-600H instrument with a data processing system. ^1^H-NMR spectra (*δ, *ppm) were recorded at 600 MHz (^13^C-NMR spectra, at 150 MHz) in DMSO-*d*_6_ using the Bruker Avance III 600 As- cend spectrometer using BBO probe. The abbreviations used in the interpretation of ^1^H NMR spectra are as follows: s, singlet; d, doublet; dd, doublet of doublets; t, triplet; br.t, broad triplet; q, quartet; m, multiplet; dist. distorted.

*Synthesis of 2-(2-amino-1,3-thiazol-4-yl)acetohydrazide (* ***2*** *)*

Ethyl 2-(2-amino-1,3-thiazol-4-yl)acetate (0.15 mol.; **1**) in methanol (60 mL) and hydrazine monohydrate (80%; 20 mL) was taken in a 500 mL round bottom flask. The reaction mixture was refluxed for 2-3 h. After complete conversion, the acid hydrazide was attained by distilling methanol off from the reaction mixture. The precipitates were filtered, washed with cold *n*-hexane, and air-dried to get purified **2**.

White crystalline solid; Yield: 90%; m.p. 170-171 ^o^C; Mol. Formula: C_5_H_8_N_4_OS; Mol. Mass.: 172 g mol^-1^; IR (KBr, ʋ, cm^-1^): 3358 (NH_2_ str.), 3351 (N-H str.), 3032 (C-H str.), 2950 (-CH_2_- str.), 1566 (C=C str.), 1587 (C=N str.), 1162 (C-N-C str.), 648 (C-S str.); ^1^H-NMR (DMSO-d_6_, 600 MHz, δ, ppm): 9.02 (br.s, 1H, 1′-CO-NH-NH_2_), 6.85 (br.s, 2H, 2-NH_2_), 6.23 (s, 1H, H-5), 4.19 (br.s, 2H, 1′-CO-NH-NH_2_), 3.19 (s, 2H, CH_2_-2′); ^13^C-NMR (DMSO-d_6_, 150 MHz, δ, ppm): 168.97 (C-1′), 168.51 (C-2), 146.43 (C-4), 102.76 (C-5), 37.32 (C-2′). Anal. Calc. for C_5_H_8_N_4_OS_2_ (172.21): C, 34.87; H, 4.68; N, 32.53. Found: C, 34.98; H, 4.84; N, 32.69; EI-MS: *m/z* 172 [M]^+^, 130 (C_4_H_6_N_2_OS)^+^, 113 (C_4_H_5_N_2_S)^+^.

*Synthesis of 2-[2-(2-amino-1,3-thiazol-4-yl)acetyl]-N-ethyl-1-hydrazinecarbothioamide (* ***4*** *)*

The hydrazide (**2**; 0.13 mol.) was dissolved in methanol (50 mL) in a 500 mL round flask by heating, and after that, ethyl isothiocyanate (0.13 mol.; **3**) was added. Reaction mixture was kept on refluxing for 1 h. After completing the reaction, precipitates of the uncyclized compound, **4,** were obtained by filtration and then dried for further use.

White amorphous solid; Yield: 86%; m.p. 176-177 ^o^C; Mol. Formula: C_8_H_13_N_5_OS_2_; Mol. Mass.: 259 gmol^-1^; IR (KBr, ʋ, cm^-1^): 3372 (N-H str.), 3052 (C-H str.), 2922 (-CH_2_- str.), 1585 (C=C str.), 1533 (C=N str.), 1158 (C-N-C bond str.), 622 (C-S str.); ^1^H-NMR (DMSO-d_6_, 600 MHz, δ, ppm): 9.87 (br.s, 1H, -CO-NH-NH-), 9.21 (br.s, 1H, -CO-NH-NH-), 7.87 (br.s, 1H, -CS-NH-), 6.88 (br.s, 2H, H_2_N-2), 6.31 (s, 1H, H-5), 3.50-3.48 (m, 4H, CH_2_-2′ & CH_2_-1′′′), 1.03 (br.t, *J* = 8.50 Hz, 3H, CH_3_-2′′′); ^13^C-NMR (DMSO-d_6_, 150 MHz, δ, ppm): 180.25 (C-1′′; HH-CS-NH), 169.05 (C-2), 168.93 (C-1′; CO-NH), 145.60 (C-4), 103.18 (C-5), 38.83 (C-1′′′), 37.36 (C-2′), 14.96 (C-2′′′). Anal. Calc. for C_8_H_13_N_5_OS_2_ (259.06): C, 37.05; H, 5.05; N, 27.00. Found: C, 37.18; H, 5.12; N, 27.13; EI-MS: *m/z *259 [M^+^], 215 (C_6_H_7_N_4_OS_2_)^+^, 141 (C_5_H_5_N_2_OS)^+^, 113 (C_4_H_5_N_2_S)^+^.

*Synthesis of 5-[(2-amino-1,3-thiazol-4-yl)methyl]-4-ethyl-4H-1,2,4-triazole-3-thiol (* ***5*** *)*

The intermediate compound (**4**; 0.13 mol.) was dissolved in 10% NaOH (100 mL) and slightly heated the solution until the compound **4** was dissolved and this solution was filtered. The precipitates of desired cyclized product, **5**, were obtained by neutralizing the filtrate of the aforementioned solution with conc. HCl in a cold state.

Light brown amorphous solid; Yield: 94%; m.p. 206-207 ^o^C; Mol. Formula: C_8_H_11_N_5_S_2_; Mol. Mass.: 241.34 gmol^-1^; IR (KBr, ʋ/cm^-1^): ʋ 3341 (N-H str.), 3062 (C-H str.), 2912 (-CH_2_- str.), 1580 (C=C str.), 1533 (C=N str.),1158 (C-N-C bond str.), 628 (C-S str.); ^1^H-NMR: δ 13.51 (s, 1H, HS-3′), 6.99 (s, 2H, H_2_N-2), 6.40 (s, 1H, H-5), 3.93 (m, 4H, CH_2_-6, CH_2_-1′′), 1.04 (t, *J* = 8.52, 3H, CH_3_-2′′); ^13^C-NMR: δ 169.22 (C-2), 166.69 (C-3′), 150.46 (C-5′), 145.55 (C-4), 103.46 (C-5), 38.92 (C-1′′), 28.26 (C-6), 13.36 (C-2′′). Anal. Calc. for C_8_H_11_N_5_S_2_ (241.34): C, 39.81; H, 4,59; N, 29.02. Found: C, 39.76; H, 4.65; N, 29.15; EI-MS: *m/z *241 [M]^+^, 139 (C_5_H_5_N_3_S)^+^, 113 (C_4_H_5_N_2_S)^+^_._

*General Synthesis of 4-({4-ethyl-5-[((un)functionalized-benzyl)sulfanyl]-4H-1,2,4-triazol-3-yl}methyl)-1,3-thiazol-2-amines (* ***7a-l*** *)*

The thiol (**5**; 0.2 g) was dissolved in DMF (3 mL) in a 100 mL round bottom flask at room temperature. After that, added one pinch of LiH and stirred it for 15-20 min. Then different un-functionalized benzyl halides **(6a-l)** were added in equimolar amounts (one in each reaction separately) and stirred for 6-8 hrs. A single spot on TLC indicated the completion of the reaction; the reaction mixture was quenched with ice cold water (50 mL). The desired derivatives, **7a-l**, were obtained through filtration or solvent extraction according to the nature of the product.

*4-{[5-(Benzylsulfanyl)-4-ethyl-4H-1,2,4-triazol-3-yl]methyl}-1,3-thiazol-2-amine (* ***7a*** *)*

Dark brown gummy liquid; Mol. Formula: C_15_H_17_N_5_S_2_; Mol. Mass.: 331 gmol^-1^. IR (KBr, ʋ, cm^-1^): 3388 (N-H str.), 3062 (C-H str. of aromatic ring), 2935 (-CH_2_- str.), 1592 (C=C str. of aromatic ring), 1522 (C=N str.), 1134 (C-N-C bond str.), 601 (C-S str.); ^1^H-NMR (600 MHz, DMSO-d_6_, δ, ppm): 7.36-7.25 (m, 5H, H-2′′′, H-3′′′, H-4′′′ H-5′′′ & H-6′′′), 6.94 (br.s, 2H, H_2_N-2), 6.23 (s, 1H, H-5), 4.34 (br.s, 2H, CH_2_-7′′′), 3.93 (br. s, 2H, CH_2_-6), 3.73 (dis. q, *J* = 6.00 Hz, 2H, CH_2_-1′′), 0.94 (dis. t, *J* = 7.14 Hz, 3H, CH_3_-2′′); ^13^C-NMR (150 MHz, DMSO-d_6_, δ, ppm): 168.56 (C-2), 153.09 (C-3′), 148.20 (C-5′), 146.17 (C-4), 137.31 (C-1′′′), 129.33 (C-2′′′ & C-6′′′), 128.65 (C-3′′′ & C-5′′′), 127.36 (C-4′′′), 102.35 (C-5), 38.45 (C-1′′), 35.14 (C-7′′′), 27.59 (C-6), 14.60 (C-2′′). Anal. Calc. for C_15_H_17_N_5_S_2_ (331.09): C, 54.35; H, 5.17; N, 21.13. Found: C, 54.65; H, 5.33; N, 21.24; EI-MS: *m/z *331 [M]^+^, 240 (C_8_H_10_N_5_S_2_)^+^, 139 (C_5_H_5_N_3_S)^+^, 113 (C_4_H_5_N_2_S)^+^, 240 (C_8_H_10_N_5_S_2_)^+^, 91 (C_7_H_7_)^+^_, _77 (C_6_H_5_)^+^.

*4-({4-Ethyl-5-[(2-methylbenzyl)sulfanyl]-4H-1,2,4-triazol-3-yl}methyl)-1,3-thiazol-2-amine (* ***7b*** *)*

Dark brown gummy liquid; Mol. Formula: C_16_H_19_N_5_S_2_; Mol. Mass.: 345 gmol^-1^. IR (KBr, ʋ, cm^-1^): 3321 (N-H str.), 3022 (C-H str. of aromatic ring), 2966 (-CH_2_- str.), 1542 (C=C str. of aromatic ring), 1528 (C=N str.), 1122 (C-N-C bond str.), 622 (C-S str.); ^1^H-NMR (600 MHz, DMSO-d_6_, δ, ppm): 7.23-7.17 (m, 4H, H-3′′′, H-4′′′, H-5′′′ & H-6′′′), 6.94 (s, 2H, H_2_N-2), 6.23 (s, 1H, H-5), 4.34 (s, 2H, CH_2_-7′′′), 3.93 (s, 2H, CH_2_-6), 3.72 (br. q, *J* = 7.20 Hz, 2H, CH_2_-1′′), 2.24 (s, 3H, CH_3_-2′′′), 0.94 (dis. t, *J* = 7.20 Hz, 3H, CH_3_-2′′); ^13^C-NMR (150 MHz, DMSO-d_6_, δ, ppm): 168.56 (C-2), 153.08 (C-3′), 148.13 (C-5′), 146.15 (C-4), 136.45 (C-1′′′), 134.74 (C-2′′′), 130.12 (C-3′′′), 127.78 (C-4′′′), 127.06 (C-6′′′), 125.86 (C-5′′′), 102.37 (C-5), 38.44 (C-1′′), 35.85 (C-7′′′), 27.57 (C-6), 18.77 (CH_3_-2′′′), 14.62 (C-2′′). Anal. Calc. for C_16_H_19_N_5_S_2_ (345.11): C, 55.62; H, 5.54; N, 20.27. Found: C, 55.68; H, 5.82; N, 20.38; EI-MS: *m/z *345 [M]^+^, 240 (C_8_H_10_N_5_S_2_)^+^, 139 (C_5_H_5_N_3_S)^+^, 113 (C_4_H_5_N_2_S)^+^, 105 (C_8_H_9_)^+^, 91 (C_7_H_7_)^+^.

*4-({4-Ethyl-5-[(3-methylbenzyl)sulfanyl]-4H-1,2,4-triazol-3-yl}methyl)-1,3-thiazol-2-amine (* ***7c*** *)*

Dark brown gummy liquid; Mol. Formula: C_16_H_19_N_5_S_2_; Mol. Mass.: 345 gmol^-1^. IR (KBr, ʋ, cm^-1^): 3338 (N-H str.), 3011 (C-H str. of aromatic ring), 2948 (-CH_2_- str.), 1572 (C=C str. of aromatic ring), 1541 (C=N str.), 1172 (C-N-C bond str.), 619 (C-S str.); ^1^H-NMR (600 MHz, DMSO-d_6_, δ, ppm): 7.13-7.06 (m, 4H, H-2′′′, H-4′′′, H-5′′′ & H-6′′′), 6.94 (br. s, 2H, H_2_N-2), 6.22 (s, 1H, H-5), 4.30 (s, 2H, CH_2_-7′′′), 3.93 (s, 2H, CH_2_-6), 3.77 (q, *J* = 7.20 Hz, 2H, CH_2_-1′′), 2.24 (s, 3H, CH_3_-3′′′), 0.94 (t, *J* = 7.38 Hz, 3H, CH_3_-2′′); ^13^C-NMR (150 MHz, DMSO-d_6_, δ, ppm): 168.56 (C-2), 153.04 (C-3′), 148.28 (C-5′), 146.18 (C-4), 137.57 (C-1′′′), 137.09 (C-3′′′), 129.42 (C-5′′′), 128.52 (C-4′′′), 128.21 (C-2′′′), 125.93 (C-6′′′), 102.32 (C-5), 39.09 (C-1′′), 37.26 (C-7′′′), 27.53 (C-6), 20.80 (CH_3_-3′′′), 14.61 (C-2′′). Anal. Calc. for C_16_H_19_N_5_S_2_ (345.11): C, 55.62; H, 5.54; N, 20.27. Found: C, 55.68; H, 5.82; N, 20.38; EI-MS: *m/z *345 [M]^+^, 240 (C_8_H_10_N_5_S_2_)^+^, 139 (C_5_H_5_N_3_S)^+^, 113 (C_4_H_5_N_2_S)^+^, 105 (C_8_H_9_)^+^, 91 (C_7_H_7_)^+^.

*4-({4-Ethyl-5-[(4-methylbenzyl)sulfanyl]-4H-1,2,4-triazol-3-yl}methyl)-1,3-thiazol-2-amine (* ***7d*** *)*

Dark brown gummy liquid; Mol. Formula: C_16_H_19_N_5_S_2_; Mol. Mass.: 345 gmol^-1^. IR (KBr, ʋ, cm^-1^): 3348 (N-H str.), 3051 (C-H str. of aromatic ring), 2931 (-CH_2_- str.), 1565 (C=C str. of aromatic ring), 1504 (C=N str.), 1139 (C-N-C bond str.), 605 (C-S str.); ^1^H-NMR (600 MHz, DMSO-d_6_, δ, ppm): 7.16 (br. d, *J* = 7.92, 2H, H-2′′′ & H-6′′′), 7.09 (br. d, *J* = 7.80, 2H, H-3′′′ & H-5′′′), 6.94 (s, 2H, H_2_N-2), 6.21 (s, 1H, H-5), 4.29 (s, 2H, CH_2_-7′′′), 3.93 (s, 2H, CH_2_-6), 3.74 (br.q, *J* = 7.30 Hz, 2H, CH_2_-1′′), 2.25 (s, 3H, CH_3_-4′′′), 0.94 (t, *J* = 7.20 Hz, 3H, CH_3_-2′′); ^13^C-NMR (150 MHz, DMSO-d_6_, δ, ppm): 168.55 (C-2), 153.01 (C-3′), 148.25 (C-5′), 146.19 (C-4), 136.62 (C-1′′′), 134.16 (C-4′′′), 129.01 (C-3′′′ & C-5′′′), 128.92 (C-2′′′ & C-6′′′), 102.31 (C-5), 38.43 (C-1′′), 37.09 (C-7′′′), 27.56 (C-6), 20.67 (CH_3_-4′′′), 14.61 (C-2′′). Anal. Calc. for C_16_H_19_N_5_S_2_ (345.11): C, 55.62; H, 5.54; N, 20.27. Found: C, 55.68; H, 5.82; N, 20.38; EI-MS: *m/z *345 [M]^+^, 240 (C_8_H_10_N_5_S_2_)^+^, 139 (C_5_H_5_N_3_S)^+^, 113 (C_4_H_5_N_2_S)^+^, 105 (C_8_H_9_)^+^, 91 (C_7_H_7_)^+^.

*4-({5-[(2-Chlorobenzyl)sulfanyl]-4-ethyl-4H-1,2,4-triazol-3-yl}methyl)-1,3-thiazol-2-amine (* ***7e*** *)*

Dark brown gummy liquid; Mol. Formula: C_15_H_16_ClN_5_S_2_; Mol. Mass.: 365 gmol^-1^. IR (KBr, ʋ, cm^-1^): 3381 (N-H str.), 3012 (C-H str. of aromatic ring), 2908 (-CH_2_- str.), 1561 (C=C str. of aromatic ring), 1547 (C=N str.), 1148 (C-N-C bond str.), 628 (C-S str.); ^1^H-NMR (600 MHz, DMSO-d_6_, δ, ppm): 7.42-7.30 (m, 4H, H-3′′′, H-4′′′, H-5′′′ & H-6′′′), 6.98 (br. s, 2H, H_2_N-2), 6.25 (s, 1H, H-5), 4.41 (s, 2H, CH_2_-7′′′), 3.94 (s, 2H, CH_2_-6), 3.75 (q, *J* = 7.20 Hz, 2H, CH_2_-1′′), 0.94 (t, *J* = 7.20 Hz, 3H, CH_3_-2′′); ^13^C-NMR (150 MHz, DMSO-d_6_, δ, ppm): 168.51 (C-2), 153.21 (C-3′), 147.65 (C-5′), 146.13 (C-4), 131.48 (C-1′′′), 129.94 (C-5′′′), 129.37 (C-3′′′), 127.55 (C-6′′′), 127.29 (C-4′′′), 127.17 (C-2′′′), 102.37 (C-5), 38.42 (C-1′′), 35.67 (C-7′′′), 27.45 (C-6), 14.54 (C-2′′). Anal. Calc. for C_15_H_16_ClN_5_S_2_ (365.05): C, 49.24; H, 4.41; N, 19.14. Found: C, 49.37; H, 4.52; N, 19.20; EI-MS: *m/z *367 [M+2]^+^, 365 [M]^+^, 240 (C_8_H_10_N_5_S_2_)^+^, 139 (C_5_H_5_N_3_S)^+^, 125 [C_7_H_6_Cl]^+^, 113 (C_4_H_5_N_2_S)^+^, 111 [C_6_H_4_Cl]^+^.

*4-({5-[(4-Chlorobenzyl)sulfanyl]-4-ethyl-4H-1,2,4-triazol-3-yl}methyl)-1,3-thiazol-2-amine (* ***7f*** *)*

Dark brown gummy liquid; Mol. Formula: C_15_H_16_ClN_5_S_2_; Mol. Mass.: 365 gmol^-1^. IR (KBr, ʋ/cm^-1^): 3355 (N-H str.), 3049 (C-H str. of aromatic ring), 2945 (-CH_2_- str.), 1592 (C=C str. of aromatic ring), 1584 (C=N str.), 1188 (C-N-C bond str.), 637 (C-S str.); ^1^H-NMR (600 MHz, DMSO-d_6_, δ, ppm): 7.49 (br. d, *J *= 8.46 Hz, 2H, H-2′′′ & H-6′′′ ), 7.32 (br. d, *J* = 8.40 Hz, 2H, H-3′′′ & H-5′′′), 6.68 (br. s, 2H, H_2_N-2), 6.29 (s, 1H, H-5), 4.34 (s, 2H, CH_2_-7′′′), 3.97 (br. s, 2H, CH_2_-6), 3.79 (q, *J* = 7.50 Hz, 2H, CH_2_-1′′), 0.91 (t, *J* = 7.20 Hz, 3H, CH_3_-2′′); ^13^C-NMR (150 MHz, DMSO-d_6_, δ, ppm): 168.82 (C-2), 152.81 (C-3′), 148.14 (C-5′), 146.17 (C-4), 136.60 (C-4′′′), 132.39 (C-1′′′), 131.01 (C-3′′′ & C-5′′′), 128.62 (C-2′′′ & C-6′′′), 102.81 (C-5), 38.51 (C-1′′), 36.28 (C-7′′′), 25.40 (C-6), 14.63 (C-2′′). Anal. Calc. for C_15_H_16_ClN_5_S_2_ (365.05): C, 49.24; H, 4.41; N, 19.14. Found: C, 49.37; H, 4.52; N, 19.20; EI-MS: *m/z *367 [M+2]^+^, 365 [M]^+^, 240 (C_8_H_10_N_5_S_2_)^+^, 139 (C_5_H_5_N_3_S)^+^, 125 [C_7_H_6_Cl]^+^, 113 (C_4_H_5_N_2_S)^+^, 111 [C_6_H_4_Cl]^+^.

*4-({5-[(2,4-Dichlorobenzyl)sulfanyl]-4-ethyl-4H-1,2,4-triazol-3-yl}methyl)-1,3-thiazol-2-amine (* ***7g*** *)*

Light brown amorphous solid; Yield: 86%; m.p.:116-117 ^o^C; Mol. Formula: C_15_H_15_Cl_2_N_5_S_2_; Mol. Mass.: 399 gmol^-1^. IR (KBr, ʋ, cm^-1^): 3362 (N-H str.), 3021 (C-H str. of aromatic ring), 2904 (-CH_2_- str.), 1543 (C=C str. of aromatic ring), 1527 (C=N str.), 1168 (C-N-C bond str.), 622 (C-S str.); ^1^H-NMR (600 MHz, DMSO-d_6_, δ, ppm): 7.63 (d, *J* = 2.00 Hz, 1H, H-3′′′), 7.38 (d, *J* = 8.20, 1H, H-6′′′), 7.33 (dd, *J* = 2.10, 8.20 Hz, 1H, H-5′′′), 6.95 (br.s, 2H, H_2_N-2), 6.23 (s, 1H, H-5), 4.39 (s, 2H, CH_2_-7′′′), 3.94 (s, 2H, CH_2_-6), 3.79 (q, *J* = 7.20 Hz, 2H, CH_2_-1′′), 0.96 (t, *J* = 7.20 Hz, 3H, CH_3_-2′′); ^13^C-NMR (150 MHz, DMSO-d_6_, δ, ppm): 168.50 (C-2), 153.26 (C-3′), 147.37 (C-5′), 145.95 (C-4), 134.00 (C-4′′′), 133.88 (C-1′′′), 133.04 (C-2′′′), 132.44 (C-6′′′), 128.82 (C-3′′′), 127.32 (C-5′′′), 102.31 (C-5), 38.47 (C-1′′), 34.65 (C-7′′′), 27.45 (C-6), 14.56 (C-2′′). Anal. Calc. for C_15_H_15_Cl_2_N_5_S_2_ (400.35): C, 45.00; H, 3.78; N, 17.49. Found: C, 45.13; H, 3.82; N, 17.61; EI-MS: *m/z *403 [M+4]^+^, 401 [M+2]^+^, 399 [M]^+^, 240 (C_8_H_10_N_5_S_2_)^+^, 159 [C_7_H_5_Cl_2_]^+^, 145 [C_6_H_3_Cl_2_]^+^, 139 (C_5_H_5_N_3_S)^+^, 113 (C_4_H_5_N_2_S)^+^.

*4-({5-[(3,4-Dichlorobenzyl)sulfanyl]-4-ethyl-4H-1,2,4-triazol-3-yl}methyl)-1,3-thiazol-2-amine (* ***7h*** *)*

Dark brown gummy liquid; Mol. Formula: C_15_H_15_Cl_2_N_5_S_2_; Mol. Mass.: 400 gmol^-1^. IR (KBr, ʋ, cm^-1^): 3366 (N-H str.), 3047 (C-H str. of aromatic ring), 2943 (-CH_2_- str.), 1608 (C=C str. of aromatic ring), 1550 (C=N str.), 1165 (C-N-C bond str.), 609 (C-S str.); ^1^H-NMR (600 MHz, DMSO-d_6_, δ, ppm): 7.63 (d, *J* = 1.92 Hz, 1H, H-2′′′), 7.55 (d, *J* = 8.28 Hz, 1H, H-5′′′), 7.24 (dd, *J* = 2.00, 8.20, 1H, H-6′′′), 6.93 (br.s, 2H, H_2_N-2), 6.21 (s, 1H, H-5), 4.35 (s, 2H, CH_2_-7′′′), 3.95 (br.s, 2H, CH_2_-6), 3.81 (q, *J* = 7.20 Hz, 2H, CH_2_-1′′), 0.97 (t, *J* = 7.10 Hz, 3H, CH_3_-2′′); ^13^C-NMR (150 MHz, DMSO-d_6_, δ, ppm): 168.58 (C-2), 153.26 (C-3′), 147.81 (C-5′), 146.15 (C-4), 133.38 (C-1′′′), 131.53 (C-4′′′), 131.07 (C-3′′′), 130.85 (C-5′′′), 130.45 (C-2′′′), 129.25 (C-6′′′), 102.31 (C-5), 38.56 (C-1′′), 35.74 (C-7′′′), 27.51 (C-6), 14.59 (C-2′′). Anal. Calc. for C_15_H_15_Cl_2_N_5_S_2_ (400.35): C, 45.00; H, 3.78; N, 17.49. Found: C, 45.13; H, 3.82; N, 17.61; EI-MS: *m/z *403 [M+4]^+^, 401 [M+2]^+^, 399 [M]^+^, 240 (C_8_H_10_N_5_S_2_)^+^, 159 [C_7_H_5_Cl_2_]^+^, 145 [C_6_H_3_Cl_2_]^+^, 139 (C_5_H_5_N_3_S)^+^, 113 (C_4_H_5_N_2_S)^+^.

*4-({5-[(2-Bromobenzyl)sulfanyl]-4-ethyl-4H-1,2,4-triazol-3-yl}methyl)-1,3-thiazol-2-amine (* ***7i*** *)*

Light brown amorphous solid; Yield: 88%; m.p.: 108-109 ^o^C; Mol. Formula: C_15_H_16_BrN_5_S_2_; Mol. Mass.: 410 gmol^-1^. IR (KBr, ʋ, cm^-1^): 3392 (N-H str.), 3074 (C-H str. of aromatic ring), 2940 (-CH_2_- str.), 1532 (C=C str. of aromatic ring), 1541 (C=N str.), 1194 (C-N-C bond str.), 625 (C-S str.); ^1^H-NMR (600 MHz, DMSO-d_6_, δ, ppm): 7.62 (dd, *J* = 0.90, 7.90 Hz, 1H, H-3′′′), 7.35 (dd, *J* = 1.50, 7.50, 1H, H-6′′′), 7.29 (dt, *J* = 1.10, 7.50 Hz, 1H, H-5′′′), 7.21 (dt, *J *= 1.70, 7.60 Hz, 1H, H-4′′′), 6.92 (br.s, 2H, H_2_N-2), 6.25 (s, 1H, H-5), 4.41 (s, 2H, CH_2_-7′′′), 3.93 (s, 2H, CH_2_-6), 3.76 (q, *J* = 7.20 Hz, 2H, CH_2_-1′′), 0.96 (t, *J* = 7.20 Hz, 3H, CH_3_-2′′); ^13^C-NMR (150 MHz, DMSO-d_6_, δ, ppm): 168.47 (C-2), 153.19 (C-3′), 147.60 (C-5′), 146.03 (C-4), 136.15 (C-1′′′), 132.65 (C-3′′′), 131.23 (C-4′′′), 129.64 (C-6′′′), 127.77 (C-5′′′), 123.73 (C-2′′′), 102.33 (C-5), 38.43 (C-1′′), 37.98 (C-7′′′), 27.46 (C-6), 14.53 (C-2′′). Anal. Calc. for C_15_H_16_BrN_5_S_2_ (410.36): C, 43.90; H, 3.93; N, 17.07. Found: C, 43.98; H, 3.99; N, 17.22; EI-MS: *m/z *411 [M+2]^+^, 409 [M]^+^, 240 (C_8_H_10_N_5_S_2_)^+^, 169 [C_7_H_6_Br]^+^, 155 [C_6_H_4_Br]^+^, 139 (C_5_H_5_N_3_S)^+^, 113 (C_4_H_5_N_2_S)^+^.

*4-({5-[(3-Bromobenzyl)sulfanyl]-4-ethyl-4H-1,2,4-triazol-3-yl}methyl)-1,3-thiazol-2-amine (****7j***) 

Bright orange amorphous solid; Yield: 84%; m.p.: 158-159 ^o^C; Mol. Formula: C_15_H_16_BrN_5_S_2_; Mol. Mass.: 4106 gmol^-1^. IR (KBr,* ʋ*, cm^-1^): 3371 (N-H str.), 3022 (C-H str. of aromatic ring), 2935 (-CH_2_- str.), 1588 (C=C str. of aromatic ring), 1562 (C=N str.), 1192 (C-N-C bond str.), 613 (C-S str.); ^1^H-NMR (600 MHz, DMSO-d_6_, δ, ppm): 7.56 (br. s, 1H, H-2′′′), 7.45 (br. d, *J* = 7.92 Hz, 1H, H-6′′′), 7.32 (br. d, *J* = 7.68 Hz, 1H, H-4′′′), 7.24 (br. t, *J* = 7.86 Hz, 1H, H-5′′′), 6.93 (br. s, 2H, H_2_N-2), 6.22 (s, 1H, H-5), 4.35 (s, 2H, CH_2_-7′′′), 3.93 (s, 2H, CH_2_-6), 3.78 (q, *J* = 7.20 Hz, 2H, CH_2_-1′′), 0.95 (t, *J* = 7.20 Hz, 3H, CH_3_-2′′); ^13^C-NMR (150 MHz, DMSO-d_6_, δ, ppm): 168.53 (C-2), 153.11 (C-3′), 147.88 (C-5′), 146.11 (C-4), 140.34 (C-1′′′), 131.57 (C-4′′′), 130.43 (C-2′′′), 130.10 (C-5′′′), 127.94 (C-6′′′), 121.34 (C-3′′′), 102.27 (C-5), 38.44 (C-1′′), 36.08 (C-7′′′), 27.44 (C-6), 14.50 (C-2′′). Anal. Calc. for C_15_H_16_BrN_5_S_2_ (410.36): C, 43.90; H, 3.93; N, 17.07. Found: C, 43.98; H, 3.99; N, 17.22; EI-MS: *m/z *411 [M+2]^+^, 409 [M]^+^, 240 (C_8_H_10_N_5_S_2_)^+^, 169 [C_7_H_6_Br]^+^, 155 [C_6_H_4_Br]^+^, 139 (C_5_H_5_N_3_S)^+^, 113 (C_4_H_5_N_2_S)^+^.

*-({5-[(4-Bromobenzyl)sulfanyl]-4-ethyl-4H-1,2,4-triazol-3-yl}methyl)-1,3-thiazol-2-amine (* ***7k*** *)*

Bright yellow amorphous solid; Yield: 90%; m.p.: 150-151 ^o^C; Mol. Formula: C_15_H_16_BrN_5_S_2_; Mol. Mass.: 410 gmol^-1^. IR (KBr, ʋ, cm^-1^): 3344 (N-H str.), 3072 (C-H str. of aromatic ring), 2927 (-CH_2_- str.), 1568 (C=C str. of aromatic ring), 1520 (C=N str.), 1135 (C-N-C bond str.), 635 (C-S str.); ^1^H-NMR (600 MHz, DMSO-d_6_, δ, ppm): 7.48 (d, *J* = 8.40, 2H, H-2′′′ & H-6′′′), 7.27 (d, *J* = 8.40 Hz, 2H, H-3′′′ & H-5′′′), 6.93 (br. s, 2H, H_2_N-2), 6.21 (s, 1H, H-5), 4.32 (s, 2H, CH_2_-7′′′), 3.93 (s, 2H, CH_2_-6), 3.76 (q, *J* = 7.20 Hz, 2H, CH_2_-1′′), 0.95 (t, *J* = 7.20 Hz, 3H, CH_3_-2′′); ^13^C-NMR (150 MHz, DMSO-d_6_, δ, ppm): 168.49 (C-2), 153.07 (C-3′), 147.87 (C-5′), 146.07 (C-4), 136.97 (C-1′′′), 131.18 (C-3′′′ & C-5′′′), 130.98 (C-2′′′ & C-6′′′), 120.44 (C-4′′′), 102.24 (C-5), 38.42 (C-1′′), 36.23 (C-7′′′), 27.46 (C-6), 14.53 (C-2′′). Anal. Calc. for C_15_H_16_BrN_5_S_2_ (410.36): C, 43.90; H, 3.93; N, 17.07. Found: C, 43.98; H, 3.99; N, 17.22; EI-MS: *m/z *411 [M+2]^+^, 409 [M]^+^, 240 (C_8_H_10_N_5_S_2_)^+^, 169 [C_7_H_6_Br]^+^, 155 [C_6_H_4_Br]^+^, 139 (C_5_H_5_N_3_S)^+^, 113 (C_4_H_5_N_2_S)^+^.

*4-({4-Ethyl-5-[(4-fluorobenzyl)sulfanyl]-4H-1,2,4-triazol-3-yl}methyl)-1,3-thiazol-2-amine (* ***7l*** *)*

Dark brown gummy liquid; Mol. Formula: C_15_H_16_FN_5_S_2_; Mol. Mass.: 349 gmol^-1^. IR (KBr, ʋ, cm^-1^): 3368 (N-H str.), 3062 (C-H str. of aromatic ring), 2947 (-CH_2_- str.), 1573 (C=C str. of aromatic ring), 1534 (C=N str.), 1122 (C-N-C bond str.), 619 (C-S str.); ^1^H-NMR (600 MHz, DMSO-d_6_, δ, ppm): 7.35-7.32 (m, 2H, H-2′′′ & H-6′′′), 7.12 (dist.t, *J* = 8.80 Hz, 2H, H-3′′′ & H-5′′′), 6.93 (s, 2H, H_2_N-2), 6.23 (s, 1H, H-5), 4.33 (br. s, 2H, CH_2_-7′′′), 3.93 (br. s, 2H, CH_2_-6), 3.75 (br. q, *J* = 7.20 Hz, 2H, CH_2_-1′′), 0.94 (t, *J* = 7.20 Hz, 3H, CH_3_-2′′); ^13^C-NMR: 168.48 (C-2), 162.13 & 162.01 (due to coupling with ^19^F, C-4′′′), 153.04 (C-3′), 147.99 (C-5′), 146.08 (C-4), 133.65 & 133.64 (C-1′′′), 130.82 & 130.80 (C-2′′′ & C-6′′′), 115.17 & 115.03 (C-3′′′ & C-5′′′), 102.28 (C-5), 38.38 (C-1′′), 36.29 (C-7′′′), 27.47 (C-6), 14.52 (C-2′′). Anal. Calc. for C_15_H_16_FN_5_S_2_ (349.08): C, 51.56; H, 4.61; N, 20.04. Found: C, 51.70; H, 4.83; N, 20.21; EI-MS: *m/z *349 [M]^+^, 240 (C_8_H_10_N_5_S_2_)^+^, 139 (C_5_H_5_N_3_S)^+^, 113 (C_4_H_5_N_2_S)^+^, 109 [C_7_H_6_F]^+^, 95 [C_6_H_4_F]^+^.

*Tyrosinase assay*

The inhibition of mushroom tyrosinase was determined by modifying the dopachrome method using L-DOPA as a substrate (54). In detail, 140 µL of phosphate buffer (20 mM, pH 6.8), 20 µL of mushroom tyrosinase (30 U/mL), and 20 µL of the inhibitor solution were placed in the wells of a 96-well microplate. After pre-incubation for 10 min at room temperature, 20 µL of L-DOPA (3,4-dihydroxyphenylalanine, Sigma Chemical, USA) (0.85 mM) was added, and the assay plate was further incubated at 25°C for 20 min. After the incubation time, the absorbance was measured at 475 nm, and the inhibition percentage was calculated related to control. Phosphate buffer and kojic acid were tested under the same conditions as the negative and positive control. The amount of inhibition by the test compounds was expressed as the percentage of concentration necessary to achieve 50% inhibition (IC_50_). Each concentration was analyzed in three independent experiments. IC_50_ values were calculated by nonlinear regression using GraphPad Prism 5.0.

The inhibition% of tyrosinase was calculated as follows:

Inhibition (%) = [(B - S)/B] × 100

Here, the B and S are the absorbance’s for the blank and samples. 

*Protocol for kinetics*

The most potent compound, **7g**, was subjected to kinetic analysis. A series of experiments were performed to determine the inhibition kinetics of **7g**, following the already reported methods (54). The concentrations for **7g** were 0.00, 0.0018, 0.0036 and 0.0072 µM. Substrate L-DOPA concentrations were between 0.0625 to 2 mM in all kinetic studies. Pre-incubation and measurement time was the same as discussed in the mushroom tyrosinase inhibition assay protocol. Maximal initial velocity was determined from the initial linear portion of absorbance up to five minutes after the addition of enzyme at the 30s interval. The inhibition type of the enzyme was assayed by Lineweaver-Burk plots of the inverse of velocities (1/*V*) versus the inverse of substrate concentration 1/[L-DOPA] mM^-1^. The EI dissociation constant *Ki *was determined by the secondary plot of 1/*V *versus inhibitor concentrations.

*Free radical scavenging assay*

Radical scavenging activity was determined by modifying the already reported method by 2, 2-diphenyl-1 picrylhydrazyl (DPPH) assay (55). The assay solution consisted of 100 µL of DPPH (150 µM), 20 µL of increasing concentration of test compounds, and the volume was adjusted to 200 µL in each well with CH_3_OH. After that, the assay reaction was then incubated for 30 minutes at room temperature. Ascorbic acid (Vitamin C) was used as a reference inhibitor. The assay measurements were carried out using a microplate reader (OPTI _Max_, Tunable) at 517 nm. The reaction rates were compared, and the percent inhibition caused by tested inhibitors was calculated. All experiments were repeated thrice.

*Computational methodology*

*Retrieval of tyrosinase in maestro*

The target protein structure was retrieved from Protein Data Bank (PDB) (www.rcsb.org) having PDBIDs 2Y9X. The protein structure was prepared using the “Protein Preparation Wizard” workflow in Schrödinger Suite. The bond orders were assigned, and hydrogen atoms were added to the protein molecule. The water molecules were removed from the protein structure. The structure was then minimized to reach the converged root mean square deviation (RMSD) of 0.30 Å with the OPLS_2005 force field. The prepared structure was employed for the further grid and docking analysis. 

*Grid generation and molecular docking*

For grid generation preparation, the active site of the tyrosinase enzyme is defined from the co-crystallized ligands from Protein Data Bank and literature data (56, 57). Grid was generated by specifying the particular residues involved in the active region of the target protein. After grid preparation, a docking experiment was performed against synthesized compounds (**7a-l**) against receptor molecules. The synthesized molecules were sketched by a 2D sketcher in the Maestro interface and utilized in docking procedure. The default docking setup parameters were employed for the ligand docking experiment (58). The predicted binding energies (docking scores) and conformational positions of the ligands within the active region of protein were also performed using the Glide experiment. Throughout the docking simulations, both partial flexibility and complete flexibility around the active site residues are achieved by Glide/SP/XP and induced fit docking (IFD) approaches (59, 60). The 3D and 2D graphical images of both best-scored docking complexes were retrieved using Maestro.

## Results and Discussion

*Chemistry*

**Scheme 1:**
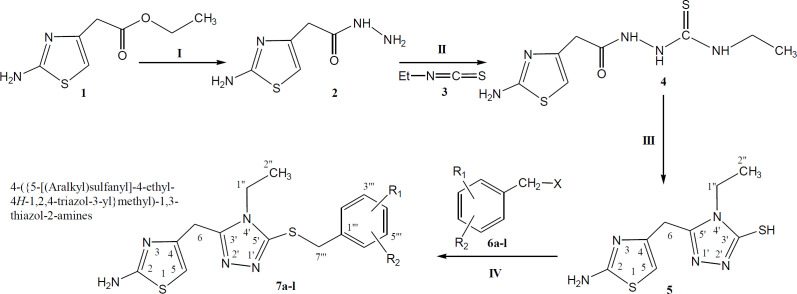
Outline for the synthesis of 4-({5-[(aralkyl)sulfanyl]-4-ethyl-4*H*-1,2,4-triazol-3-yl}methyl)-1,3-thiazol-2-amines. Reagents and Conditions: (I) MeOH/N_2_H_4_•H_2_O/refluxing for 2 hrs. (II) MeOH/Refluxing for 1 hr. (III) The ppt. of **4** dissolved by slightly heating in 10% NaOH/filtration/acidification of filtrate in cold state to get ppt. of **5**. (IV) DMF/LiH/stirring for 12-24 h

The synthesis of designed *S*-aralkylated bi-heterocyclic hybrid molecules has been outlined in (Scheme 1), and the varying groups are listed in (Table 1). The convergent synthetic process was carried out by refluxing ethyl 2-(2-amino-1,3-thiazol-4-yl)acetate (**1**) with hydrazine in methanol to get 2-(2-amino-1,3-thiazol-4-yl)acetohydrazide (**2**). The precipitates of **2** were collected, by filtration, and it was further refluxed with ethyl isothiocyante (**3**) in methanol to obtain an intermediary compound, 2-[2-(2-amino-1,3-thiazol-4-yl)acetyl]-*N*-ethyl-1-hydrazinecarbothioamide (**4**) which was cyclized to get a solid nucleophile, 5-[(2-amino-1,3-thiazol-4-yl)methyl]-4-ethyl-4*H*-1,2,4-triazole-3-thiol (**5**). This bi-heterocyclic nucleophile (**5**) was dissolved in DMF, and one pinch of LiH was added. The solution was stirred for 15-20 minutes to activate the mercapto position of this molecule. Then, in the last step, it was treated with equimolar amounts of various aralkyl halides (**6a-l**), acting as electrophiles, to acquire the targeted hybrid molecules (**7a-l**).

**Figure 2:**
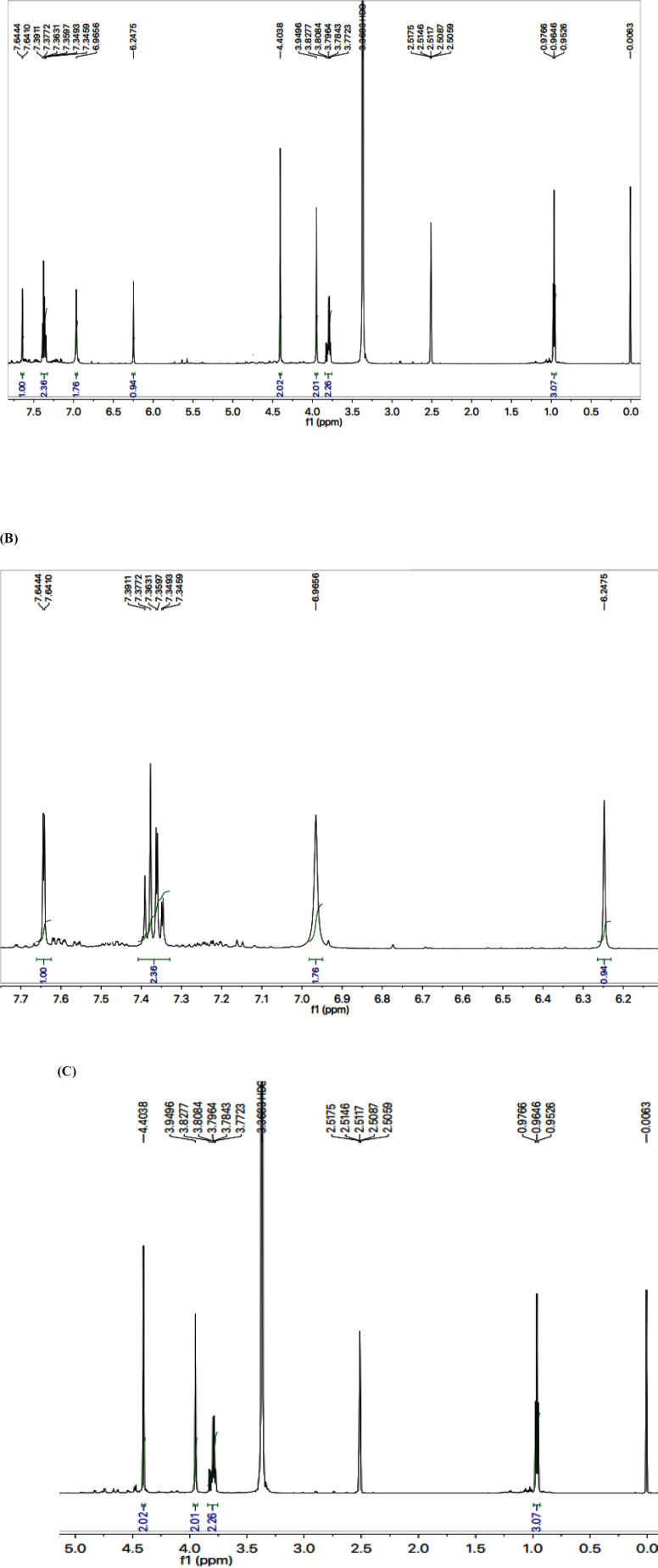
**(**A) ^1^H-NMR spectrum of **7g**. (B) Expanded aromatic region of ^1^H-NMR spectrum of **7g**. (C) Expanded aliphatic region of ^1^H-NMR spectrum of **7g**

The structure analysis of one of the compounds is elaborated hereby in detail for the benefit of the reader. Compound **7g** was synthesized as a light brown amorphous solid with a melting point of 116-117 ^o^C, and its molecular formula, C_15_H_15_Cl_2_N_5_S_2_, was corroborated by the molecular ion peak at *m/z* 399 along with CHN analysis data. The count of the number of protons in its ^1^H-NMR spectrum and the number of carbon resonances in its ^13^C-NMR spectrum was also coherent with this assignment. The functional groups were ascertained by its IR spectrum, whereby the characteristic peaks appeared at 3362 (N-H stretching), 3021 (C-H stretching of aromatic ring), 2904 (-CH_2_- stretching), 1543 (C=C stretching of aromatic ring), 1527 (C=N stretching), 1168 (C-N-C bond stretching), 622 (C-S stretching), indicating the presence of heterocyclic rings. In ^1^H-NMR spectrum, two peculiar signals of an ethyl group, attached to a nitrogen atom of triazole ring, appeared at *δ* 3.79 (q, *J* = 7.20 Hz, 2H, CH_2_-1′′) and 0.96 (t, *J* = 7.20 Hz, 3H, CH_3_-2′′), while an AMX spin system of 2,4-dichlorobenzyl group was signified by three typical signals in aromatic region at *δ* 7.63 (d, *J* = 2.00 Hz, 1H, H-3′′′), 7.38 (d, *J* = 8.20, 1H, H-6′′′) and 7.33 (dd, *J* = 2.10, 8.28 Hz, 1H, H-5′′′). The benzylic methylene, attached with sulfur atom, appeared at *δ* 4.39 (s, 2H, CH_2_-7’’’). 2-Amino-1,3-thiazol-4-yl heterocycle was categorized by two signals at *δ* 6.95 (br. s, 2H, H_2_N-2) and 6.23 (s, 1H, H-5) while the signal *δ* 3.94 (s, 2H, CH_2_-6) was assignable to a methylene group connecting the two heterocycles in the molecule. ^1^H-NMR spectrum of this molecule has been shown in Figure 2A while Figure 2B displayed the expanded aromatic region. The expanded aliphatic part of this spectrum has been shown in Figure 2C. 

**Figure 3:**
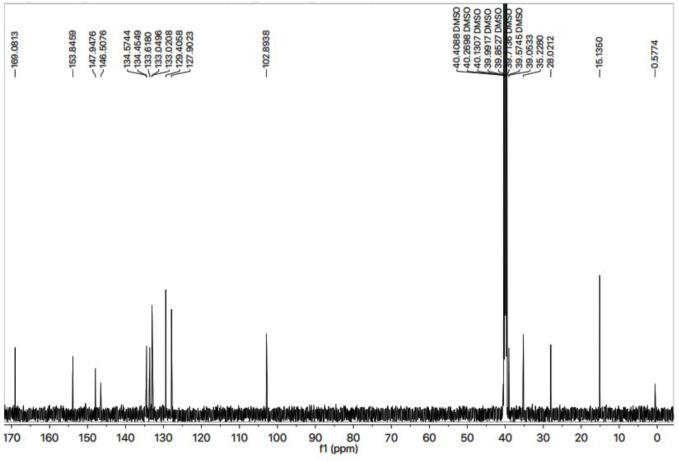
^13^C-NMR spectrum of **7g**

**Figure 4:**
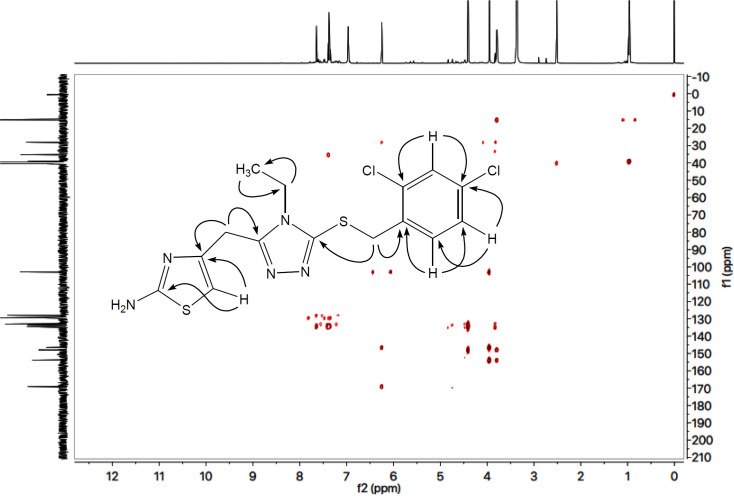
HMBC spectrum of **7g **along with significant correlations

All these assignments are also verified by its ^13^C-NMR spectrum (Figure 3), which exhibited overall fifteen carbon resonances. 2-Amino-1,3-thiazol-4-yl heterocycle was clearly specified by two quaternary signals at δ 168.50 (C-2) and 145.95 (C-4) along with a methine signal at δ 102.31 (C-5). Likewise, the other heterocycle *i.e*. (1,2,4-triazol-5-yl)sulfanyl was also indicated by two quaternary signals at *δ* 153.26 (C-3’) and 147.37 (C-5’). The methylene connecting the two heterocycles was apparent at *δ* 27.45 (C-6). The 2,4-dichlorobenzyl moiety was also obvious with three quaternary signals at *δ* 134.00 (C-4’’’), 133.88 (C-1’’’) and 133.04 (C-2’’’) along with three methine signals at *δ* 132.44 (C-6’’’), 128.82 (C-3’’’) and 127.32 (C-5’’’), in addition to a distinct signal at *δ 34.65 (C-7*’’’) for a benzylic methylene, attached to sulfur atom. The ethyl group connected with the triazole ring was also designated by one methylene signal at *δ 38.47 (C-1*’’) and one methyl signal at *δ 14.66 (C-2*’’). The C-H connectivities in the carbon skeleton were thoroughly corroborated by its HMBC spectrum, and the important correlations are illustrated on this spectrum (Figure 4). These spectral data thoroughly confirmed the structure of this molecule and was named as 4-({5-[(2,4-dichlorobenzyl)sulfanyl]-4-ethyl-4*H*-1,2,4-triazol-3-yl}methyl)-1,3-thiazol-2-amine. Likewise, the structures of all other compounds were characterized by their spectral data.

*Biology*

*Tyrosinase inhibition and structure-activity relationship*

**Figure 5:**
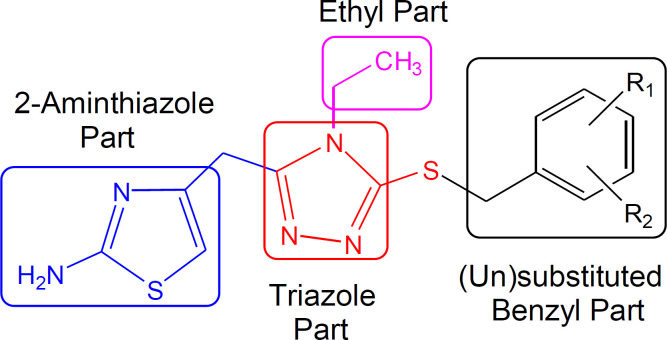
General structural parts of compounds, **7a-l**

The ethylated bi-heterocyclic hybrids (**7a-l**) were investigated against tyrosinase enzyme to explore their inhibitory potentials, and the results obtained so are tabulated (Table 2). These compounds exposed very persuasive inhibitory activities against this enzyme, which was apparent from their lower IC_50 _(µM) values, relative to the standard (Kojic acid), having IC_50_ value of 16.8320 ± 1.1600 µM. Although the displayed activity is characteristic of the whole molecule, a limited structure-activity relationship (SAR) was anticipated by perceiving the effect of varying (un) substituted-benzyl entities on the inhibitory potential. This approximation was made because it was the only part which was varying in all molecules. The general structural parts of the studied compounds are demarcated in (Figure 5).

**Figure 6:**
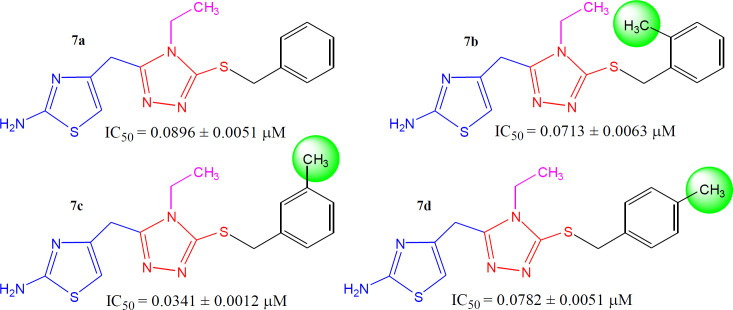
Structure-activity relationships of compounds, **7a**, **7b**, **7c**, and **7d**

The methylated molecules, **7b**, **7c**, and **7d,** exhibited greater inhibitory potential relative to **7a** (IC_50_ = 0.0896 ± 0.0051 *µ*M), having unsubstituted-benzyl part. However, among the methylated *regio*-isomers, the compound **7c** with *meta*-methyl group was found to be superb inhibitor (IC_50_ = 0.0341 ± 0.0012 *µ*M), as compared to *ortho*-isomer (**7b**, IC_50_ = 0.0713 ± 0.0063 *µ*M), as well as *para*-isomer (**7d**, IC_50_ = 0.0782 ± 0.0051 *µ*M). It means, when a small-sized electron-donating group was present at 3-position in benzylic part (Figure 6), the molecule was prone to make some superior interactions with the enzyme. 

**Figure 7:**
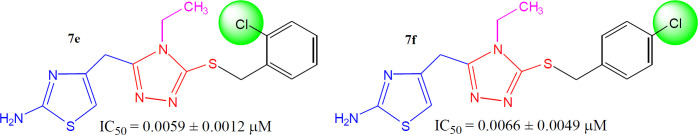
Structure-activity relationship of compounds, **7e** and **7f**

The *mono*-chloro derivatives possessed very resembling inhibitory activities (Figure 7). It means, whether a medium-sized dual-natured group is present at *ortho*-position (**7e**, IC_50_ = 0.0059 ± 0.0012 *µ*M) or at *para*-position (**7f**, IC_50_ = 0.0066 ± 0.0049 *µ*M), the molecules render some analogous interactions to the enzyme. 

**Figure 8.**
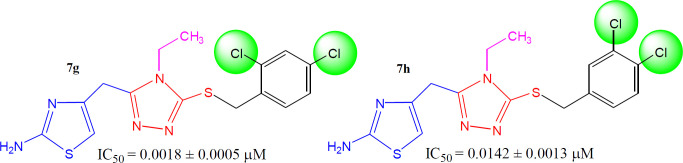
Structure-activity relationship of compounds, **7e** and **7f**

In between the dichloro-isomers, the compound **7g** (IC_50_ = 0.0018 ± 0.0005 *µ*M), having *ortho* and *para*-positioned chloro groups behaved as a superb inhibitor than **7h** (IC_50_ = 0.0142 ± 0.0013 *µ*M) in which the two chloro groups were present at adjacent positions* i.e. meta* and *para* (Figure 8). Moreover, **7g** was also identified as the best inhibitor among the whole synthetic series, indicating the suitability of medium-sized dual natured chloro groups at 2 and 4-position in benzylic part to inhibit tyrosinase. 

**Figure 9:**
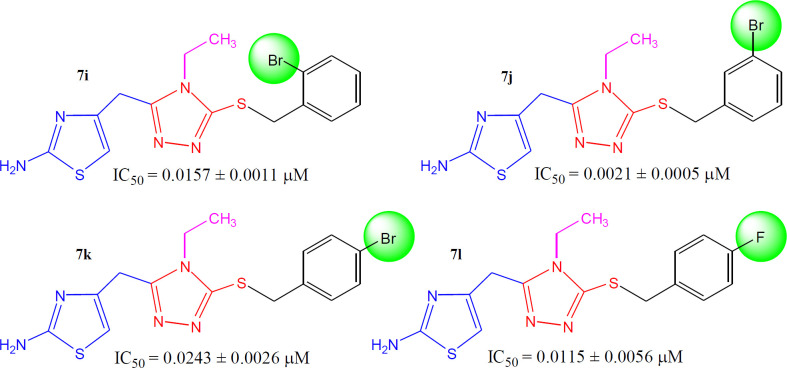
Structure-activity relationships of compounds, **7i, 7j, 7k** and **7l**

When the inhibitory potential of fluoro and bromo derivatives was compared, it was observed that the presence of a bulky bromo group at *meta*-position (in **7j**) was a suitable option. Furthermore, this compound was also recognized as the second most active molecule (IC_50_ = 0.0021 ± 0.0013 *µ*M) in the synthetic series. The shifting of bromo group from *ortho* position (**7i**, IC_50_ = 0.0157 ± 0.0011 *µ*M) to *para* position (**7k**, IC_50_ = 0.0243 ± 0.0026 *µ*M) resulted in a decrease in the activity. Likewise, the presence of a highly electronegative fluoro group in **7h** (IC_50_ = 0.0115 ± 0.0056 *µ*M) was also not a superior choice (Figure 9). 

 So, it was inferred from the structure-activity relationship that among such bi-heterocyclic hybrids, the molecules with mono-chloro groups or bromo group at *meta*-position or di-chloro derivatives having *ortho* and *para* substituents in benzylic part are generally suitable entities for the promising inhibition of tyrosinase enzyme.

*Kinetic analysis*

**Figure 10:**
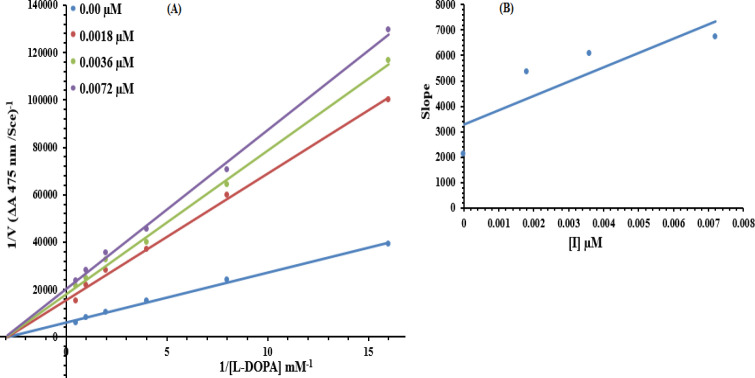
Lineweaver–Burk plots for inhibition of tyrosinase in the presence of compound **7g**. (A) Concentrations of **7g** were 0.00, 0.0018, 0.0036 and 0.0072 µM, respectively. Substrate L-DOPA concentrations were 0.0625, 0.125, 0.25, 0.5, 1 and 2 mM, respectively. (B) The insets represented the plot of the slope versus inhibitor **7g** concentrations to determine the inhibition constant. The lines were drawn using linear least squares fit

Based upon our results, the most potent compound **7g** was selected to determine their inhibition type and inhibition constant on tyrosinase. The potential of these compounds to inhibit free enzyme and enzyme-substrate complex was determined in terms of EI and ESI constants, respectively. The kinetic studies of the enzyme by the Lineweaver-Burk plot of 1/V versus 1/[S] in the presence of different compounds’ concentrations gave a series of straight lines (Figure 10A). The results of **7g** showed that this compound intersected within the second quadrant. The analysis showed that V_max_ decreased to new increasing doses of inhibitors; on the other hand, K_m_ remained the same. This behavior indicated that compound **7g** inhibited the tyrosinase non-competitively from forming the enzyme-inhibitor complex. The Secondary plot of slope against the concentration of inhibitors showed enzyme inhibitor dissociation constant (K_i_) (Figure 10B). The kinetic results are presented in Table 3.

*Free radical scavenging*

**Figure 11:**
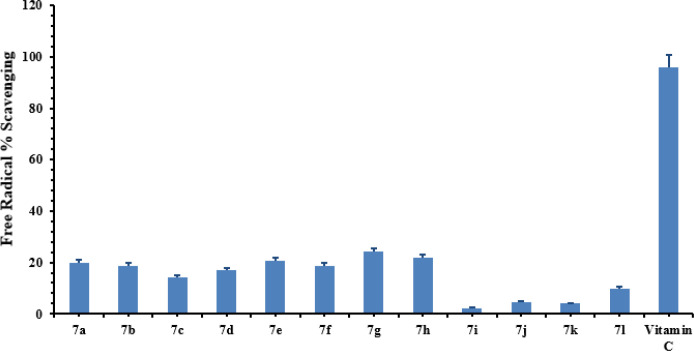
Free radical% scavenging activity of synthetic compounds. The values were represented as mean ± SEM (Standard error of the mean). The concentration of all compounds was 100 µg/mL

The antioxidant potential of all synthesized compounds, **7a-g**, has been determined using Ascorbic acid (Vitamin C) as a reference to compare the antioxidant activity of the synthesized compounds. The results obtained from DPPH assay have been presented (Figure 11), whereby it was found that the synthesized compounds exhibited low to moderate antioxidant activity. The compound **7h**, bearing 3,4-dichlorobenzyl group, exhibited 21% radical scavenging activity while **7g**, having a 2,4-dichlorobenzyl group, showed 24% radical scavenging potential. In general, most of the compounds exhibited very feeble antioxidant activities relative to that of standard Vitamin C.

*Molecular docking and binding energy analyses*

Molecular docking is a significant approach to study the interactive behavior of newly synthesized ligands within the active region of target proteins (61, 62). The docked complexes of synthesized compounds, **7a-l**, against mushroom tyrosinase were analyzed based on the lowest binding energy values (kcal/mol) and hydrogen/hydrophobic interaction pattern. Results showed that all the ligands, **7a-l**, exhibited good docking energy values and showed their interaction within the active region of the target protein. GlideScore is based on ChemScore (fitness function), but includes a steric-clash term, adds buried polar terms devised by Schrödinger to penalize electrostatic mismatches. The GScore is calculated from the Equation 1.

GScore = vdW + Coul + Lipo + Hbond + Metal + BuryP + RotB + Site Equation 1.

vdW = Van der Waals energy, Coul = Coulomb energy, Lipo = Lipophilic, Hbond = Hydrogen-bonding, Metal = Metal-binding, BuryP = Penalty for buried polar groups, RotB = Penalty for freezing rotatable bonds and Site = Polar interactions in the active site. 

**Figure 12:**
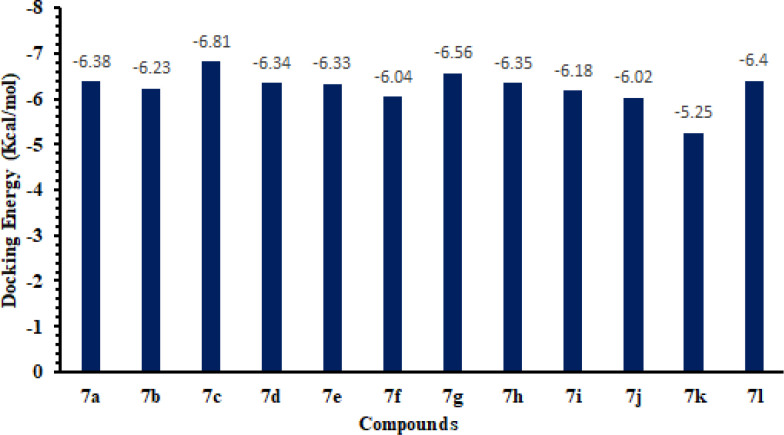
Docking energy values of synthesized compounds

Based on *in-vitro* and *in-silico* docking energy results, **7c** was ranked as the best ligands, which showed good inhibitory potential against targeted enzymes as compared to all other derivatives. Although the basic nucleus of all the synthesized compounds was the same, most compounds possess good efficient energy values and have no big energy fluctuations difference. The docking binding energy values are depicted here (Figure 12).

*Binding pocket and ligands binding conformations*

**Figure 13:**
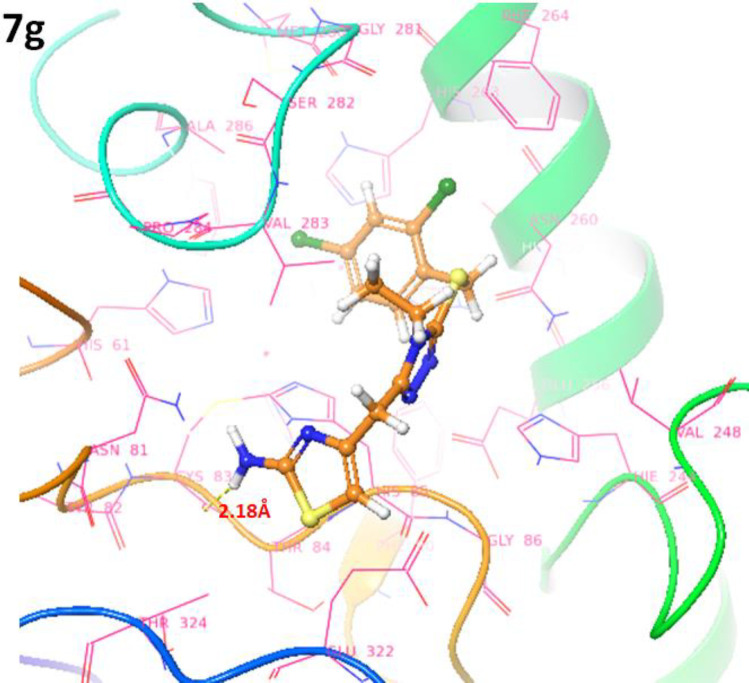
Docking complexes of 7g. The ligand structures 7g is highlighted in brown color while the interactive residues are depicted in pink color

**Figure 14:**
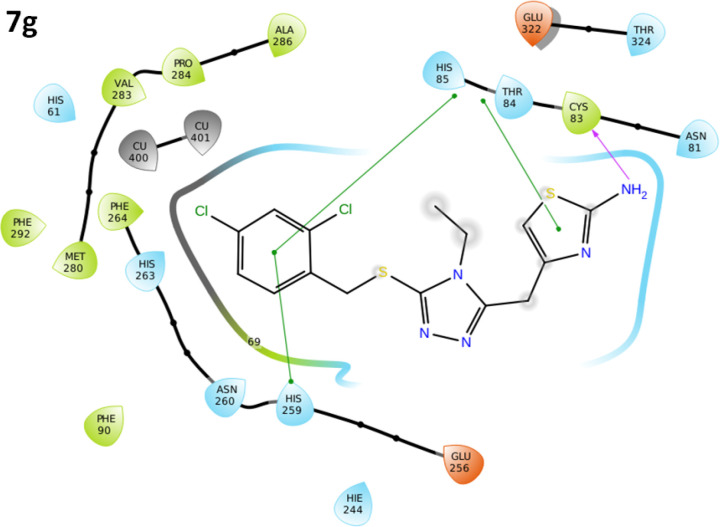
2D-docking complexes of **7g**

The binding pocket analysis showed that ligands, **7a-l**, were confined in the active region of the target protein. Results showed that all the synthesized compounds were bound in the binding pocket, having an appropriate conformational pattern. The docked complexes were analyzed based on bonding interactions pattern. Based on *in-vitro* and docking energy results, the most promising compound (**7g**) was selected to check its binding and conformational position within the active region of the target protein (Figures 13 and 14). The 2-amino group of thiazole ring formed hydrogen bond with Cys83 having bond length 2.18 Å. It has been observed that hydrogen bond length should be less than 3.5 Å in docking complexes. Our results showed that the bond length was comparable with the standard value. Moreover, π-π interactions were observed between the thiazole ring and aromatic residue His85. The di-choloro benzyl group formed a couple of the hydrophobic interactions at His85 and His259 residues within the active region of the target protein with appropriate bond distances. Both residues are metal-bound residues and play a significant role in protein functionality. The already published data showed a good correlation with our docking results, strengthening our docking reliability (56, 57). The 2-dimensional graphical depiction of all other docking complexes is in supplementary file (Figures S1-S13). 

**Table 1:** Different groups (-R_1_ and -R_2_) in Scheme 1

**Compd.**	**-R**_1_	**-R**_2_
**6a, 7a**	-H	-H
**6b, 7b**	2-CH_3_	-H
**6c, 7c**	3-CH_3_	-H
**6d, 7d**	-H	4-CH_3_
**6e, 7e**	2-Cl	-H
**6f, 7f**	-H	4-Cl
**6g, 7g**	2-Cl	4-Cl
**6h, 7h**	3-Cl	4-Cl
**6i, 7i**	2-Br	-H
**6j, 7j**	3-Br	-H
**6k, 7k**	-H	4-Br
**6l, 7l**	-H	4-F

**Table 2:** Tyrosinase inhibitory activity of ethylated bi-heterocyclic hybrids, **7a-l**.

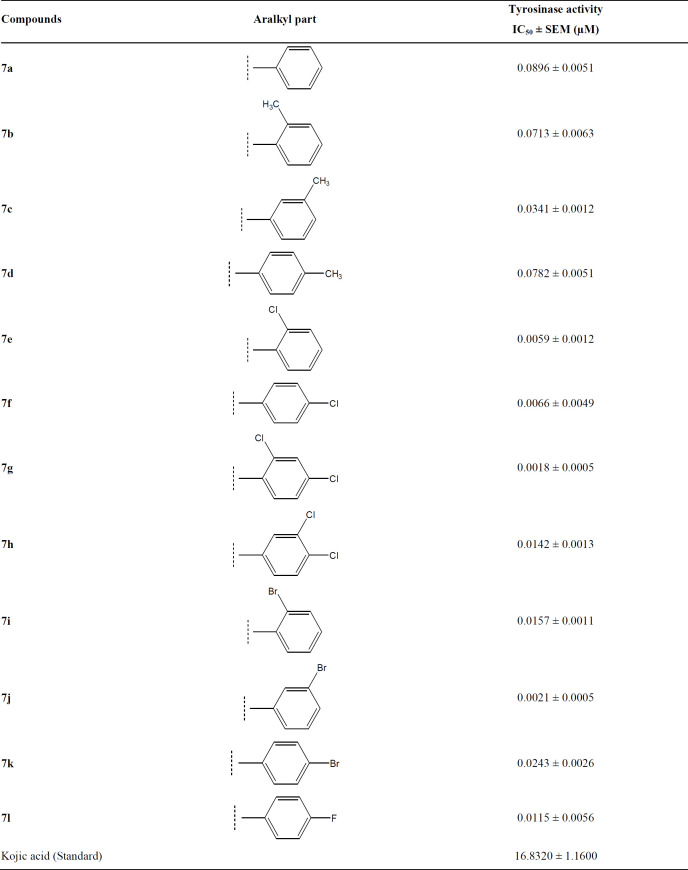

SEM = Standard error of the mean; values are expressed in mean ± SEM.

**Table 3:** Kinetic parameters of the mushroom tyrosinase for L-DOPA activity in the presence of various concentrations of **7g**

**Concentration (µM)**	**V**_max _ **(ΔA/s)**	**K**_m_ ** (mM)**	**Inhibition Type**	**K**_i_ **(µM)**
0.00	0.00017	0.3	Non-Competitive	0.0057
0.0018	6.67578 × 10^-5^	0.3		
0.0036	4.71212 × 10^-5^	0.3		
0.0072	4.22262 × 10^-5^	0.3		

V_max_ is the reaction velocity, K_m_ is the Michaelis-Menten constant, and K_i_ is the EI dissociation constant.

## Conclusion

In conclusion, a new series of bi-heterocyclic hybrids were synthesized as propitious tyrosinase inhibitors. Significantly, the compounds bearing 3-bromobenzyl, 2-chlorobenzy, 4-chlorobenzyl, or 2,4-dichlorobenzyl groups possessed very superb activities. Moreover, molecular docking results also revealed good binding interactions and docking energy values. Therefore, it was concluded generally that these bi-heterocyclic molecules might be deliberated as commendable medicinal scaffolds for treating tyrosinase related ailments, particularly skin disorders.

## Supplementary Materials


