# Supplementary material for: Synthesis, Kinetics, Binding Conformations and Structure-activity Relationship of Potent Tyrosinase Inhibitors: Aralkylated 2-aminothiazole-ethyltriazole Hybrids
Source: Iran J Pharm Res. 2021 Spring;20(2):206–28. doi: 10.22037/ijpr.2020.15521.13145 (PMC8457735; doi:10.22037/ijpr.2020.15521.13145)
Supplement: Supplementary file 1 [file ijpr-20-206-s001.pdf]

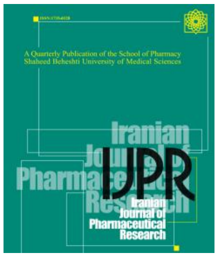

Supplementary Materials for

Synthesis, Kinetics, Binding Conformations and Structure-activity Relationship of Potent Tyrosinase Inhibitors: Aralkylated 2-Aminothiazole-Ethyltriazole Hybrids

Abdul Rehman Sadiq Butt, Muhammad Athar Abbasi*, Aziz-ur-Rehman, Sabahat Zahra Siddiqui, Hussain Raza, Mubashir Hassan, Syed Adnan Ali Shah and Sung-Yum Seo

*To whom correspondence should be addressed. E-mail: abbasi@gcu.edu.pk

Volume 20, Issue 2 (Spring 2021)

This PDF file includes:
Figures S1-S11

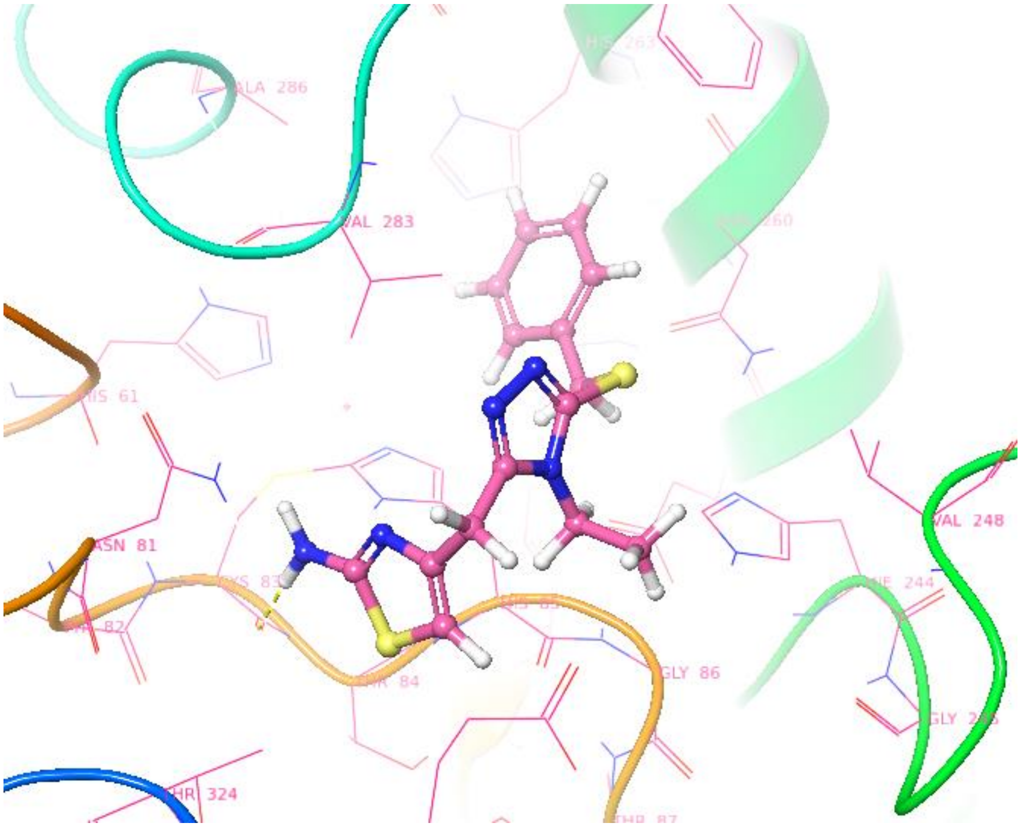

Figure S1. Docking complex of **7a.**

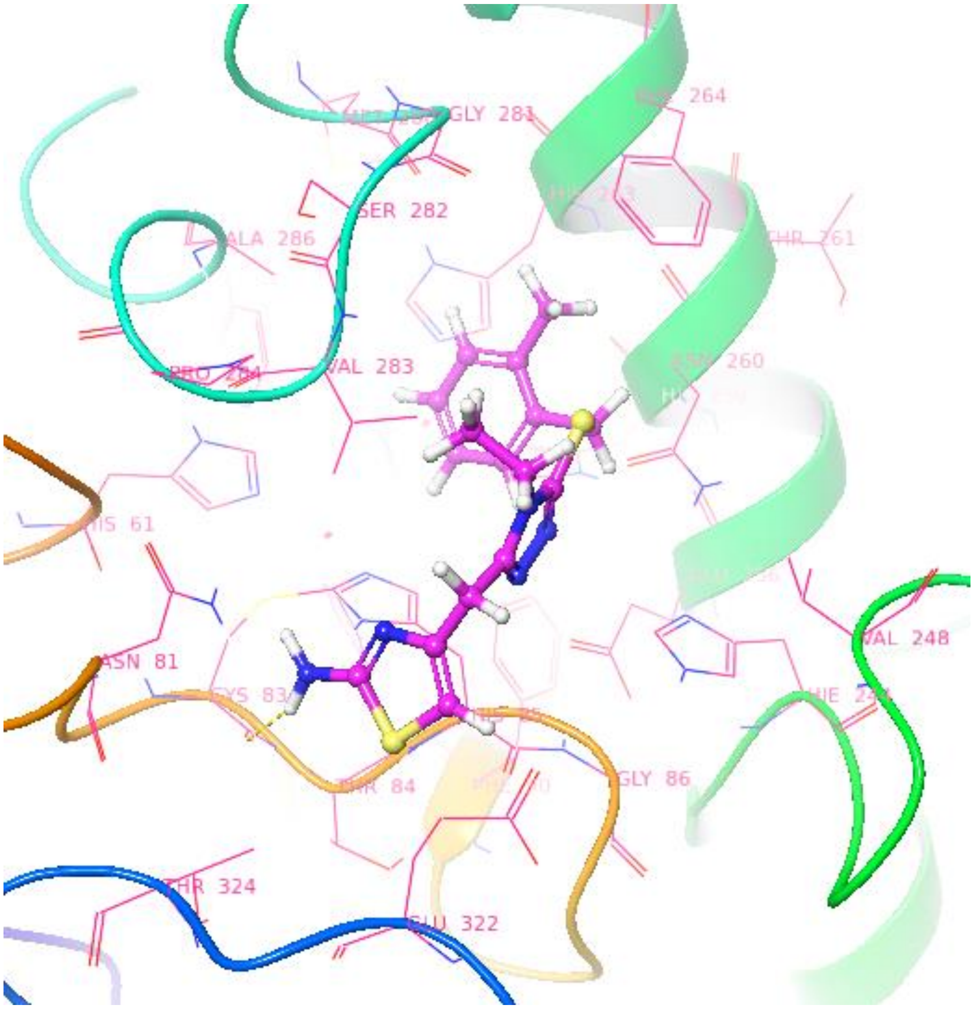

Figure S2. Docking complex of **7b**.

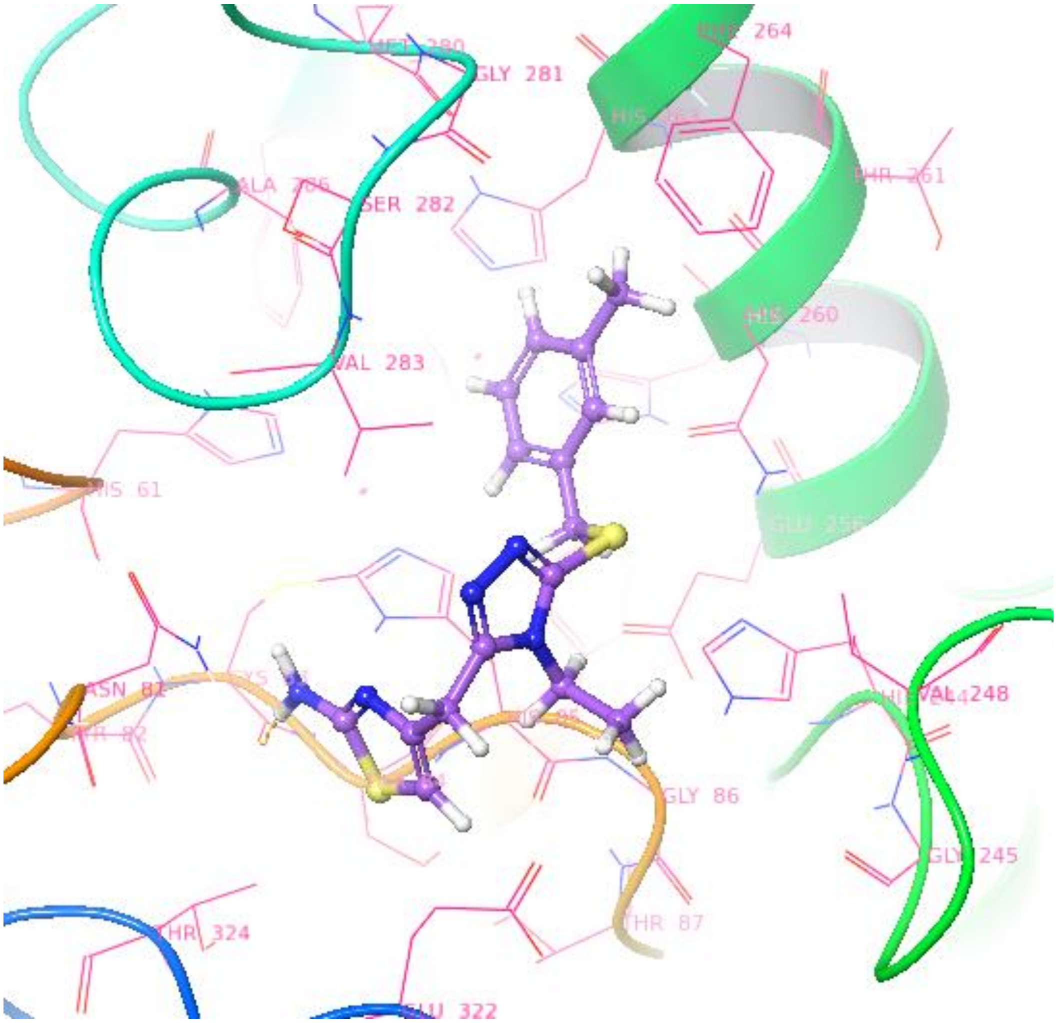

Figure S3. Docking complex of **7c**.

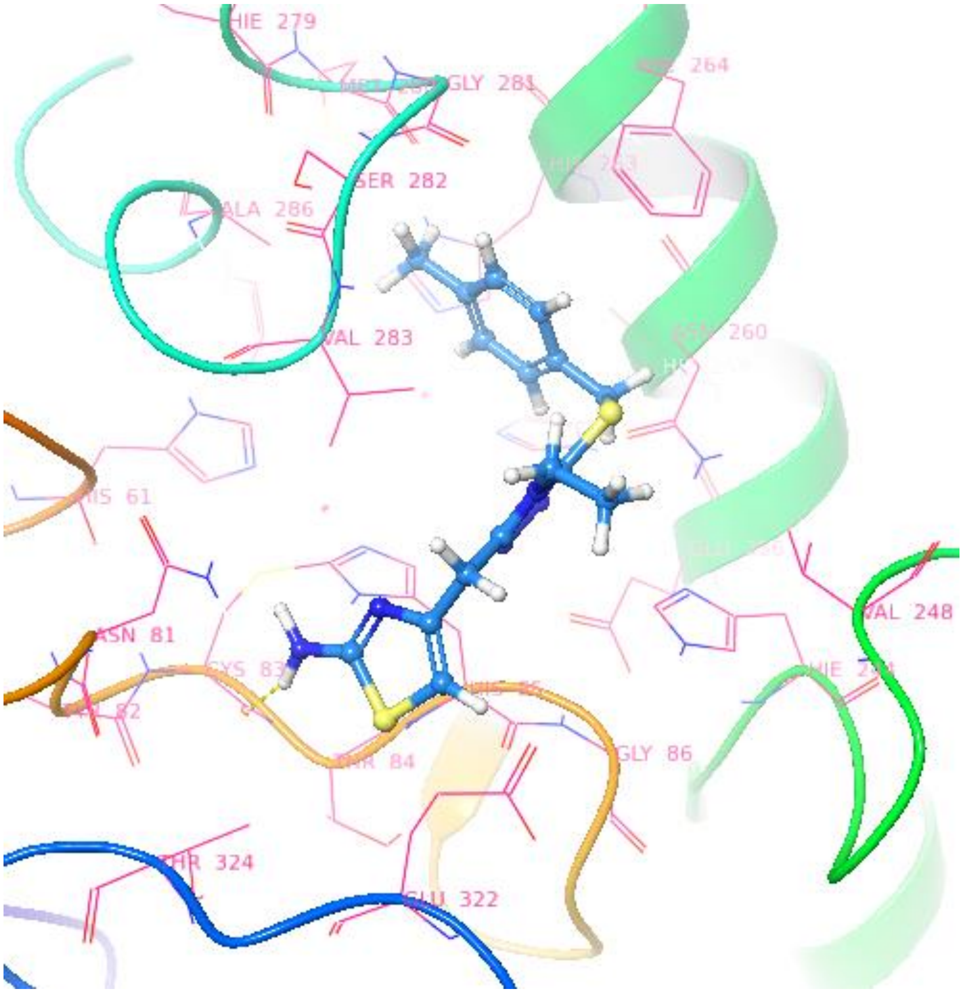

Figure S4. Docking complex of **7d**.

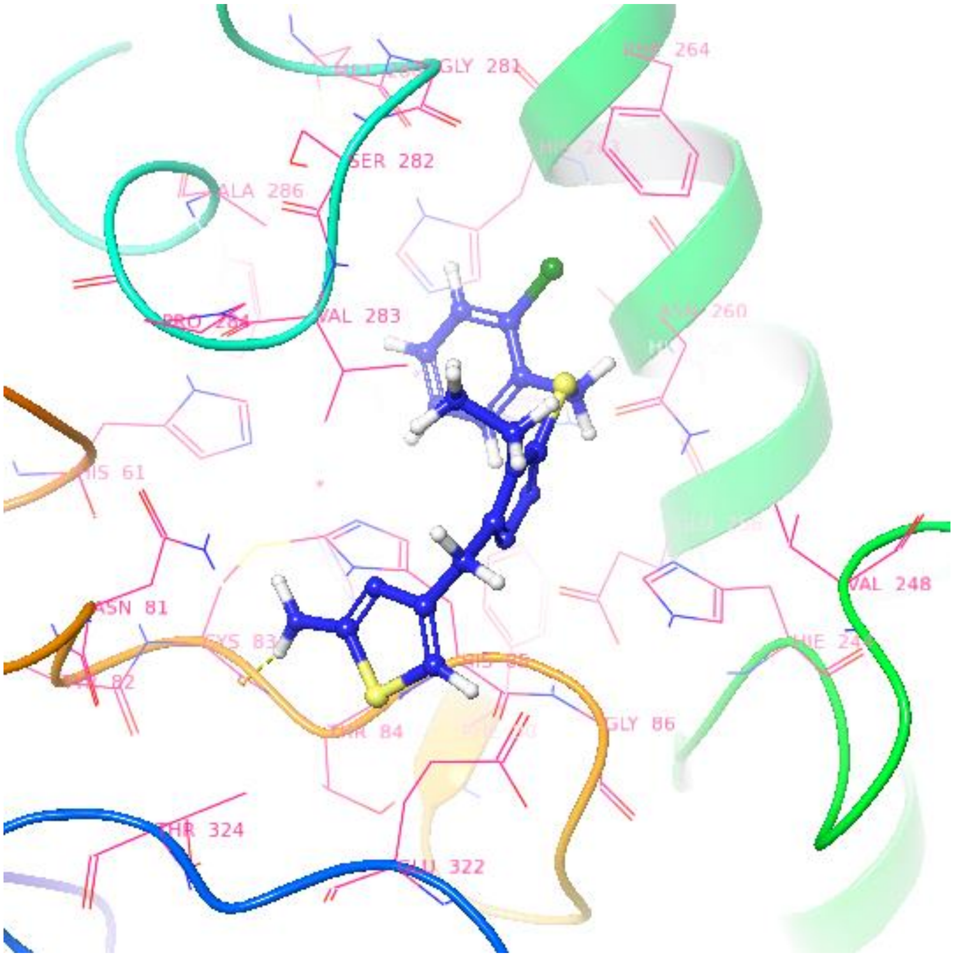

Figure S5. Docking complex of **7e**.

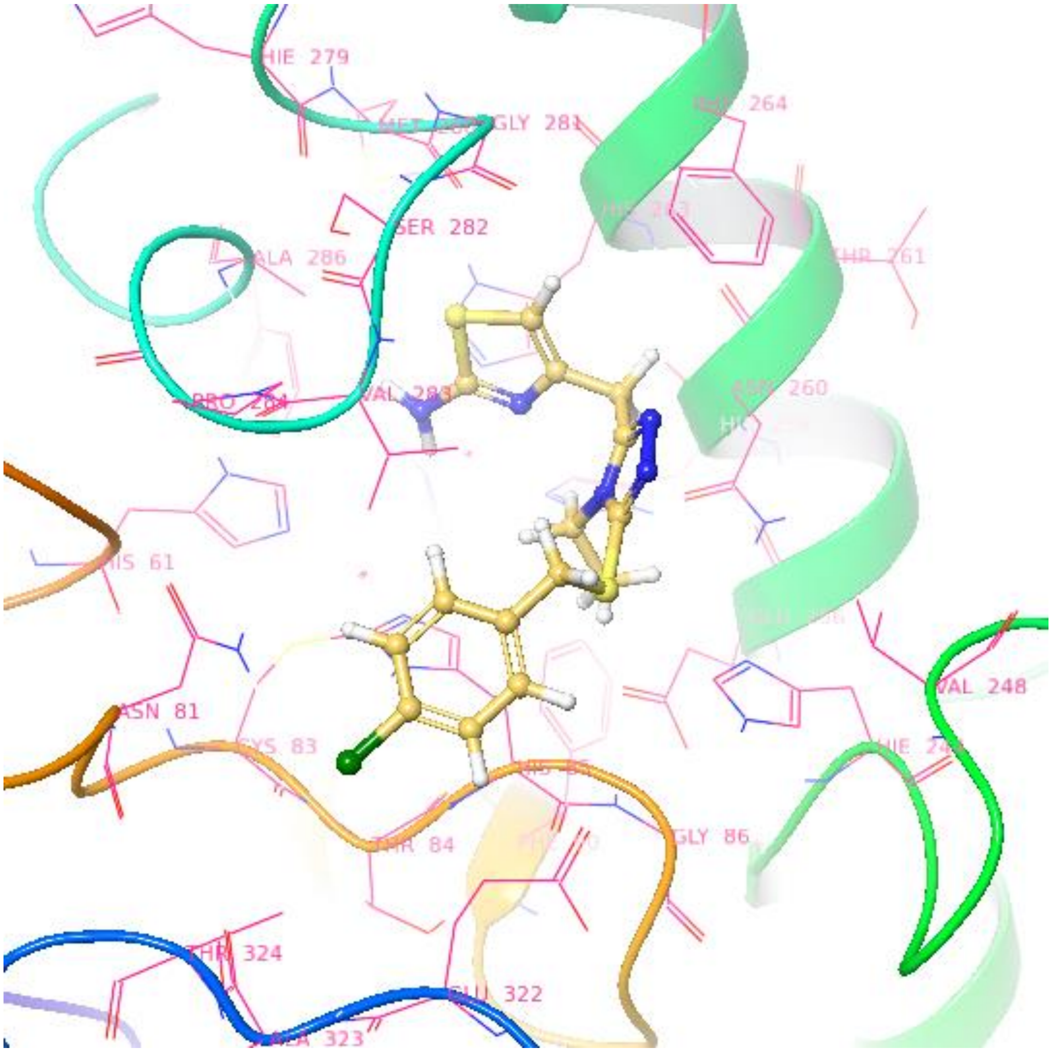

Figure S6. Docking complex of **7f**.

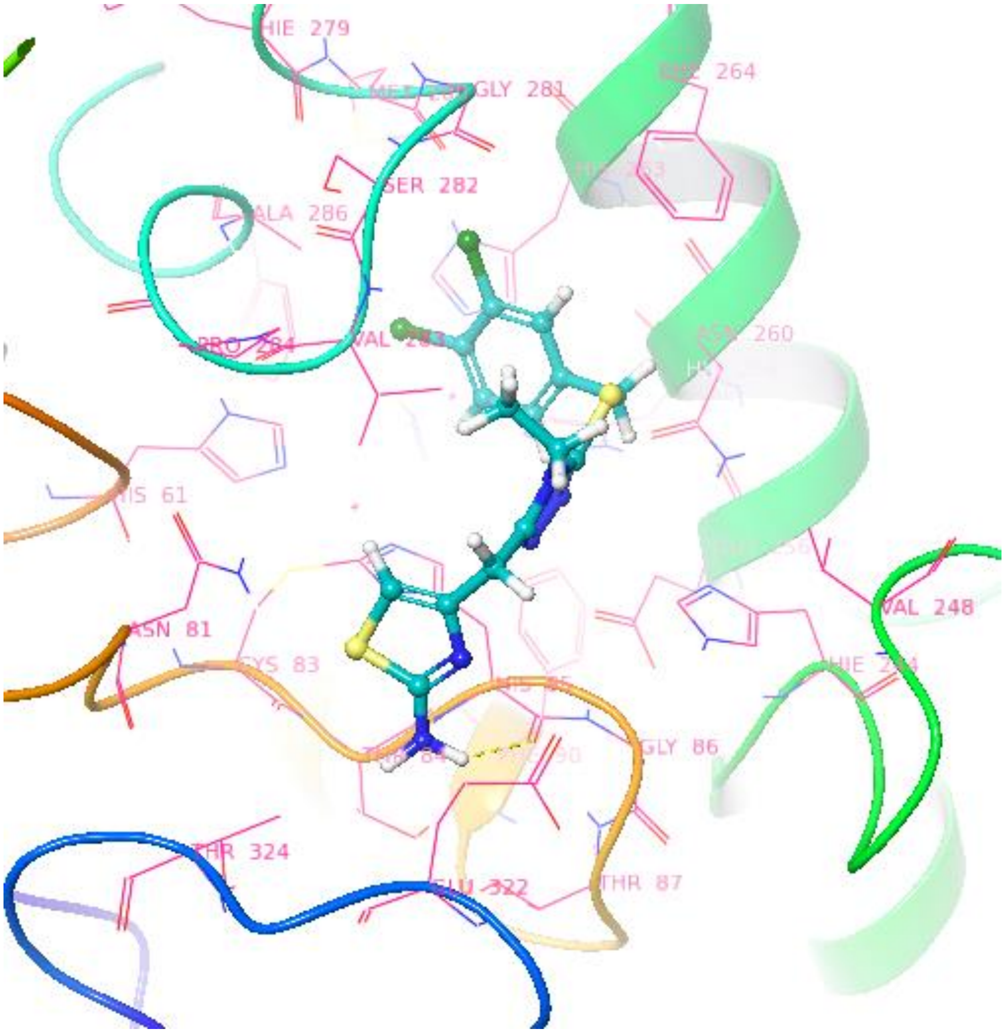

Figure S7. Docking complex of **7h**.

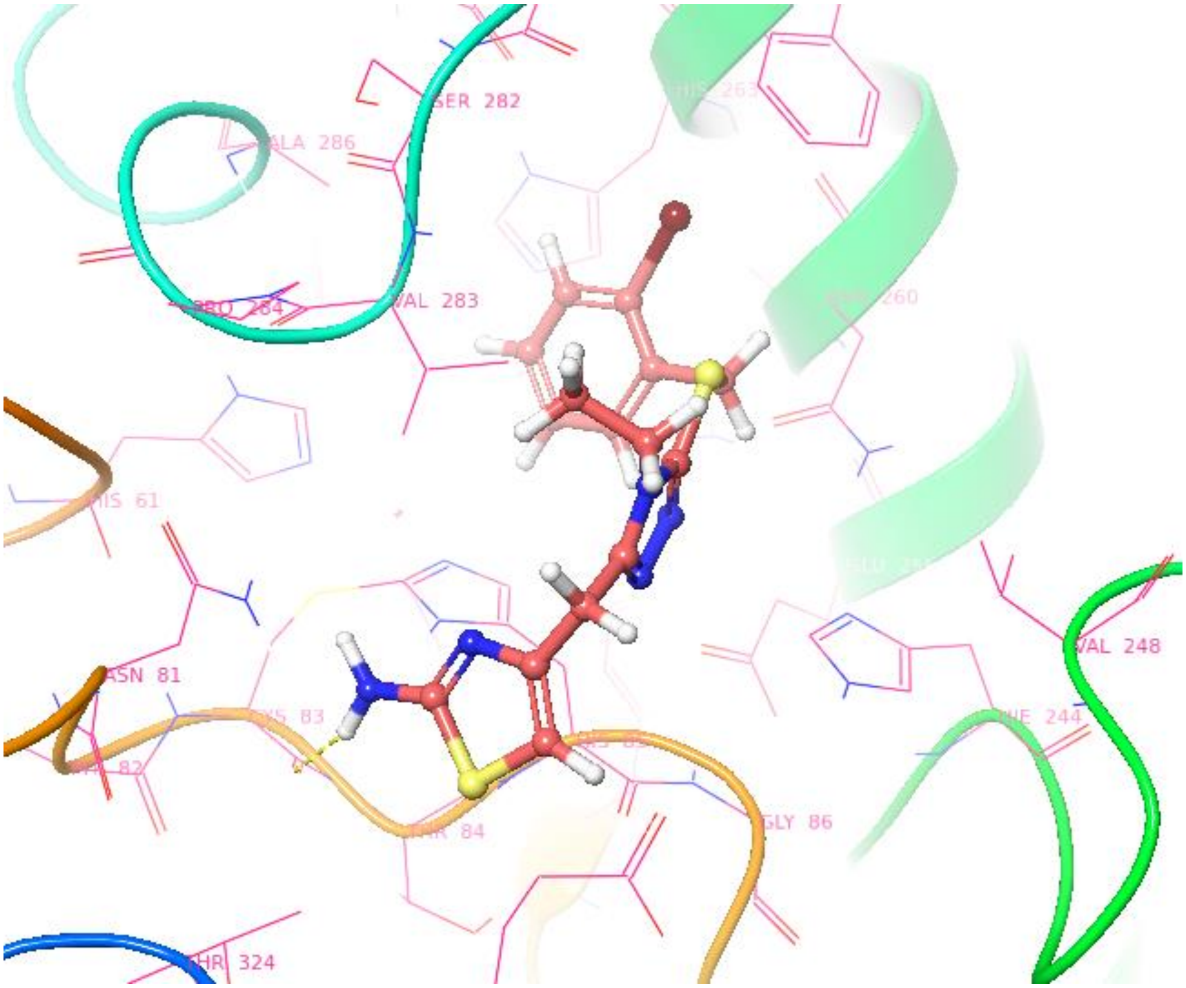

Figure S8. Docking complex of **7i**.

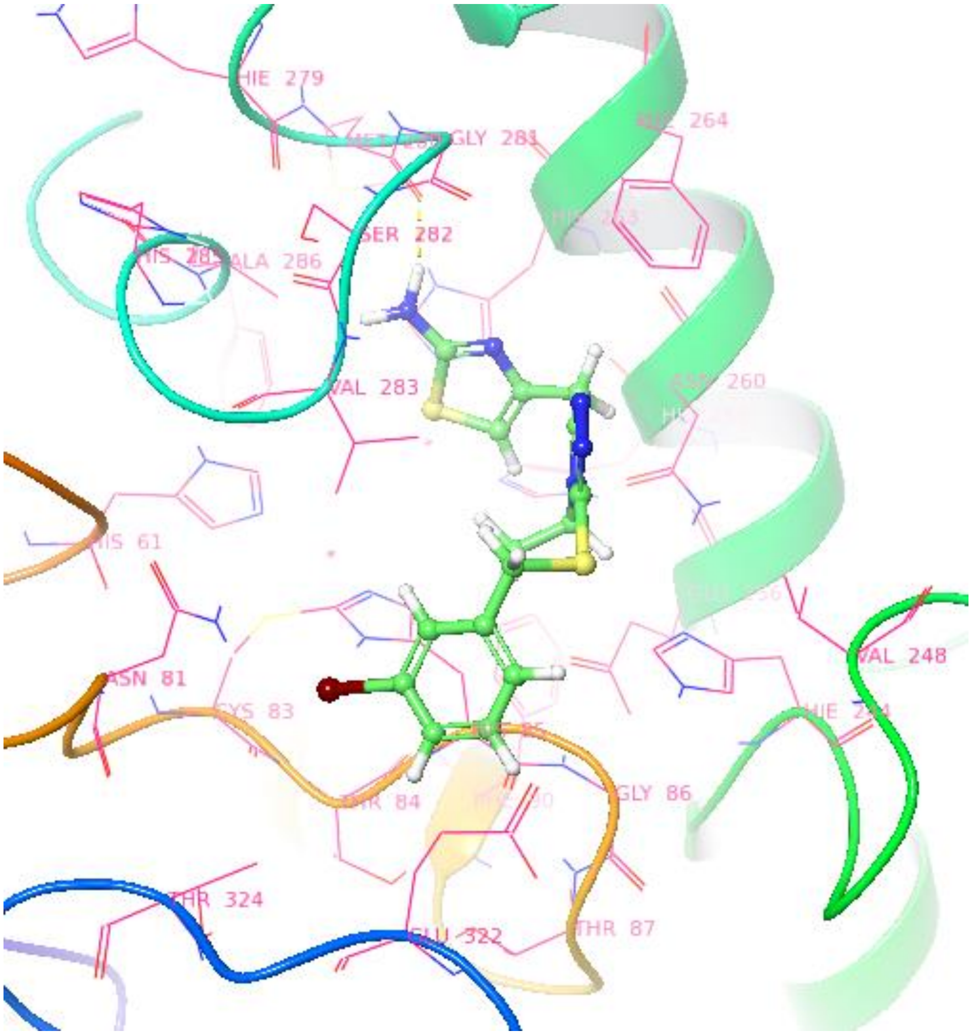

Figure S9. Docking complex of **7j**.

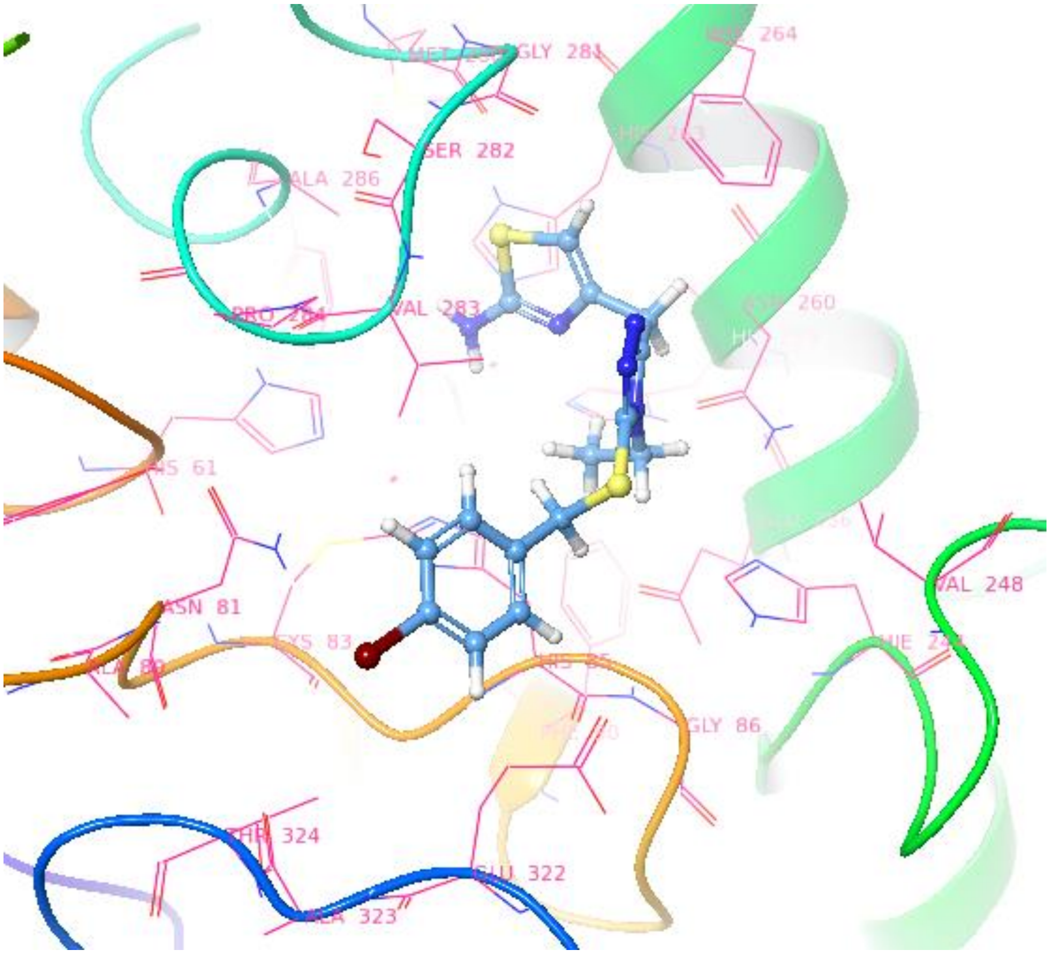

Figure S10. Docking complex of **7k**.

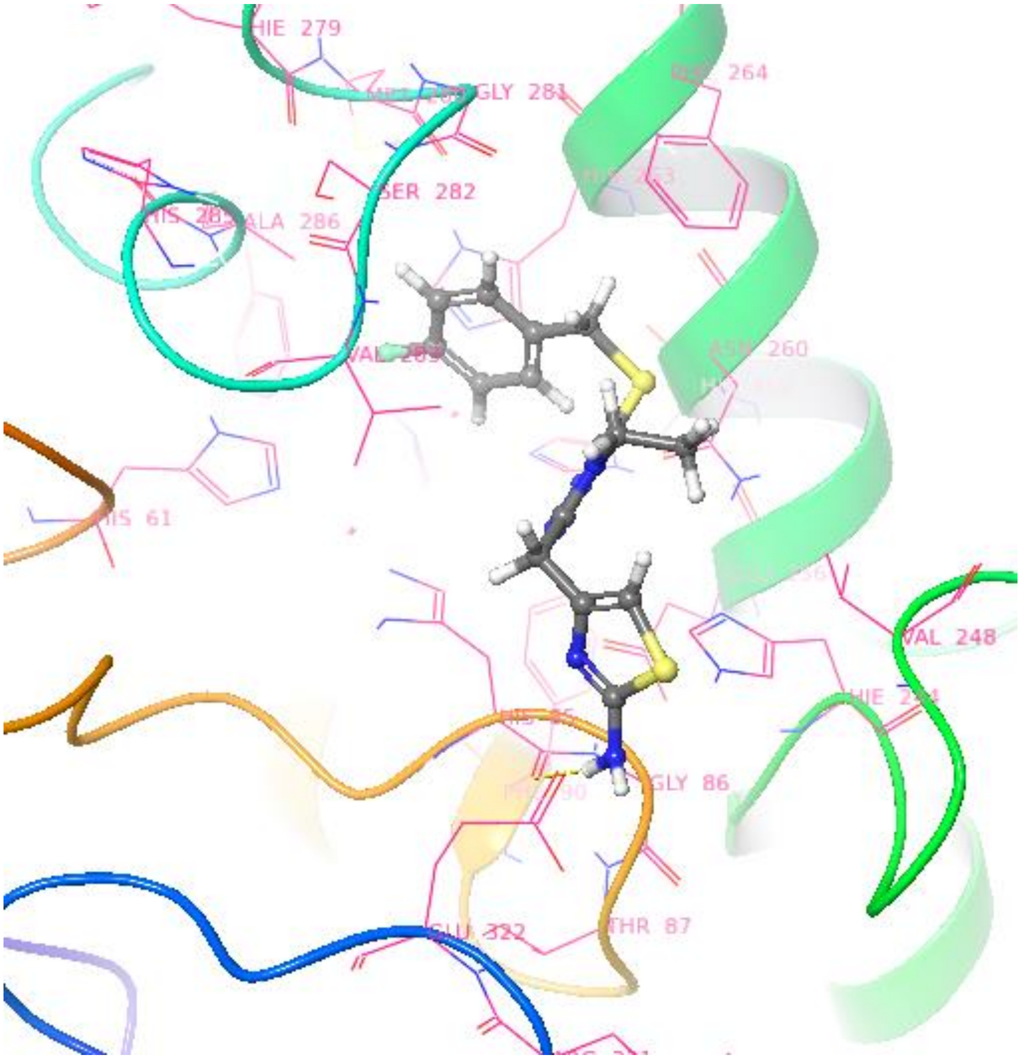

Figure S11. Docking complex of **7l**.
